# Mercury records from natural archives reveal ecosystem responses to changing atmospheric deposition

**DOI:** 10.1093/nsr/nwae417

**Published:** 2024-11-19

**Authors:** Qinqin Chen, Qingru Wu, Yuying Cui, Shuxiao Wang

**Affiliations:** School of Environment, Tsinghua University, Beijing 100084, China; State Key Joint Laboratory of Environmental Simulation and Pollution Control, Beijing 100084, China; State Environmental Protection Key Laboratory of Sources and Control of Air Pollution Complex, Beijing 100084, China; School of Environment, Tsinghua University, Beijing 100084, China; State Key Joint Laboratory of Environmental Simulation and Pollution Control, Beijing 100084, China; State Environmental Protection Key Laboratory of Sources and Control of Air Pollution Complex, Beijing 100084, China; School of Environment, Tsinghua University, Beijing 100084, China; State Key Joint Laboratory of Environmental Simulation and Pollution Control, Beijing 100084, China; State Environmental Protection Key Laboratory of Sources and Control of Air Pollution Complex, Beijing 100084, China; School of Environment, Tsinghua University, Beijing 100084, China; State Key Joint Laboratory of Environmental Simulation and Pollution Control, Beijing 100084, China; State Environmental Protection Key Laboratory of Sources and Control of Air Pollution Complex, Beijing 100084, China

**Keywords:** mercury pollution, natural archive, GEOS-Chem, ecosystem recovery, policy evaluation

## Abstract

Global ecosystems face mercury contamination, yet long-term data are scarce, hindering understanding of ecosystem responses to atmospheric Hg input changes. To bridge the data gap and assess ecosystem responses, we compiled and compared a mercury accumulation database from peat, lake, ice and marine deposits worldwide with atmospheric mercury deposition modelled by GEOS-Chem, focusing on trends, magnitudes, spatial–temporal distributions and impact factors. The mercury fluxes in all four deposits showed a 5- to 9-fold increase over 1700–2012, with lake and peat mercury fluxes that generally mirrored atmospheric deposition trends. Significant decreases in lake and peat mercury fluxes post-1950 in Europe evidenced effective environmental policies, whereas rises in East Asia, Africa and Oceania highlighted coal-use impacts, *inter alia*. Conversely, mercury fluxes in marine and high-altitude ecosystems did not align well with atmospheric deposition, emphasizing natural influences over anthropogenic impacts. Our study underscores the importance of these key regions and ecosystems for future mercury management.

## Teaser

This study compiled a global mercury accumulation database from natural deposits and compared it with modelled atmospheric deposition, revealing significant regional differences in mercury flux trends. This study highlights the importance of targeted environmental policies for effective mercury management in the future.

## INTRODUCTION

Mercury (Hg) is recognized as one of the top 10 global pollutants due to its high toxicity and strong tendency to bioaccumulate in the environment [1]. Mercury is mobilized by anthropogenic activities such as metal mining and fossil-fuel burning [2,3], and natural activities such as volcanic eruptions and biomass burning, as well as re-emissions from legacy Hg [1]. Emitted Hg primarily exists in a gaseous form (Hg^0^) that can travel long distances. During transport, Hg^0^ may be oxidized to Hg^2+^ and methylmercury, both of which are more bio-accumulative and water-soluble. This transported Hg is subsequently deposited into terrestrial and marine ecosystems through dry and wet deposition processes, leading to contamination [4]. Once deposited, Hg eventually accumulates in environmental compartments such as aquatic sediments and peat [1], posing long-term risks to ecosystems and human health [5]. To mitigate the adverse effects of Hg, the Minamata Convention on Mercury—an international legally binding treaty—came into force in 2017 [6]. This convention complements national atmospheric protection policies that could synergistically reduce Hg emissions through end-of-pipe controls. Notable examples of these policies include the UK’s Clean Air Act of 1956 and the USA’s Clean Air Act of 1970, which are among the earliest regulations for controlling air pollution.

Due to these pollution control efforts, recent observations have shown reduced Hg emissions and ambient concentrations in the Arctic [7], Europe and North America [8–10]. However, these reductions in emissions and ambient concentrations might not fully indicate changes in contamination levels within underlying surface ecosystems. Ecosystems include various elements such as organisms, waterbodies and natural deposits, each governed by unique Hg deposition mechanisms. Of particular concern and research interest are natural deposits, including peat, lake sediments, marine sediments and ice, as they serve as the final Hg sinks and potential Hg sources of the respective ecosystems. These natural deposits inherently preserve and accumulate environmental contaminants such as Hg in chronological order [11–13] and thus are known as natural archives. In particular, the Hg that has accumulated in nature archives in undisturbed regions was considered to be primarily sourced from atmospheric depositions [14]. Therefore, such long-term natural archive Hg records are valuable for studying how respective ecosystems—particularly their natural deposit component—respond to changing atmospheric Hg deposition.

Different types of natural archives may not respond in the same way to changing atmospheric inputs due to unique Hg deposition processes. For instance, Hg in peat is primarily from atmospheric deposition, which encompasses vegetation uptake through active absorption by plant roots and foliage [15–19] and is influenced by peat growth and microbial decomposition [20]. In lake sediments, Hg accumulates from direct atmospheric deposition including vegetation fixation and catchment runoff, and legacy Hg from catchment soil [21–23]. Marine sediments acquire Hg through a balance of atmospheric deposition and re-emissions, with waterbodies [24] and sea ice as natural barriers to Hg exchange [25]. Coastal erosion can also contribute to Hg inputs into marine sediments [26]. In ice sheets and glaciers, Hg accumulates primarily through atmospheric deposition. Significant photoreduction of Hg [27], along with ice sublimation and melting [27–29], contributes to annual Hg deposition loss. Table S1 summarizes that major depositional processes of Hg to peat, lake sediments, marine sediments and ice. Previous studies reviewed Hg records from natural archives such as peat and lake sediments and offered qualitative assessments at regional and hemispheric scales [30–33] (Text S1). However, these studies were limited in providing quantitative comparisons across ecosystems and regions. Such comparisons would be invaluable for understanding different ecosystem responses, evaluating the effectiveness of source-control policies and informing future mitigation strategies.

In this study, we aim to utilize the Hg records from natural archives along with atmospheric modelling to understand how different ecosystems—at least their natural deposit components acting as the Hg sinks—respond to changing atmospheric Hg deposition. First, we compiled a natural archive Hg database from 1700 to 2012. The database consisted of Hg accumulation fluxes of 221 cores extracted from ice, peat, lake and marine deposits. These deposits, primarily influenced by atmospheric Hg deposition, were sampled from eight key regions worldwide, covering the period from 1700 to 2012. Second, we compared the trends, rates and magnitudes of Hg accumulation in four natural archives throughout 1980–2012 with the respective atmospheric Hg deposition modelled by GEOS-Chem. This comparison elucidated how Hg levels in these archives respond to atmospheric changes, thereby enhancing our understanding of Hg records across eight key regions. Last, based on the unique responses of natural archives across regions, we discussed policy effectiveness and highlighted key regions and ecosystems that may require more targeted Hg management strategies.

## RESULTS AND DISCUSSION

### Natural archive mercury database

**Figure 1.**
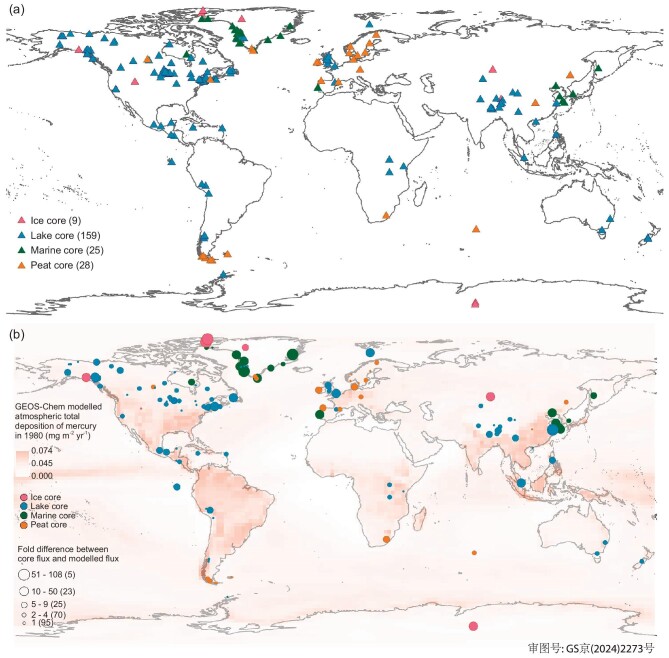
(a) Spatial distributions of natural archive records of Hg. The numbers in brackets represent the number of cores from the respective natural archives compiled in the database. Note that some cores collected from the same or nearby locations are not fully visible in the figure; please refer to Dataset S1 for detailed core information. (b) A comparison between natural archive Hg fluxes and total (wet + dry) atmospheric Hg deposition fluxes modelled by GEOS-Chem at each coring site in the base year 1980—a year with the greatest number of cores. Larger circles indicate greater disparities in magnitude. Generally, lake-Hg, peat-Hg and marine-Hg fluxes are greater than the modelled total atmospheric Hg deposition fluxes, while ice-Hg fluxes are smaller. A total of 42% of the cores show good agreement with the modelled values, indicated by a difference within 1-fold. However, 12% of the cores exhibit differences of >10-fold, mostly marine and ice cores.

We meticulously selected 221 cores that were primarily impacted by atmospheric Hg deposition, as indicated in the respective literature (Fig. 1a; see detailed methods in Text S2, Figs S1–S3 and Dataset S1). The core selection was based on five stringent criteria, including the requirements that the cores should be free from significant physical and chemical disturbances, provided Hg accumulation flux (mg m^−2^ yr^−1^) and covered the period from 1700 to 2012 with a temporal resolution finer than 20 years, considering potential chronological errors. These selected cores were categorized into eight key regions, with the highest number of cores from North America (47%), followed by Europe (11%), the Arctic (11%, mainly Greenland), Latin America (9%, mainly Central America and the western Andes), Central Asia (8%, mainly Tibetan Plateau), East Asia (6%), Central and Southern Africa (3%) and Oceania (2%) (see Table S2 for a full list). In terms of core types, 72% were lake cores (distributed globally), followed by peat cores (13%, mainly in Europe), marine cores (11%, in continental shelf areas) and ice cores (4%, in polar and mountainous regions).

Cores from different studies exhibit varying temporal scales, which can significantly impact the accuracy of regionally synthesized data. This inconsistency affects the precision of annually averaged Hg accumulation flux data within each region. To resolve this issue, we employed the General Additive Model (GAM), which offers flexibility by allowing for non-linear relationships between predictors and the response variable [34]. This approach enabled the prediction of Hg accumulation fluxes up to 2012 for cores whose uppermost layers did not date to the year 2012, thereby ensuring temporal consistency and enhancing the accuracy and reliability of our Hg flux predictions. We fed GAM with eight selected predictors (detailed in Table S4): local anthropogenic emission, local non-anthropogenic emission (i.e. natural emissions and re-emissions), global total emission, surface temperature, precipitation, greenness fraction, elevation (or depth for marine cores) and the ratio between catchment area and lake area (applicable only for lake cores). The GAM analysis established correlations between these predictors and Hg accumulation fluxes in each of the four types of natural archives. The GAM result showed that the four correlations explained 65%, 88%, 85% and 83% of the deviances in lake, peat, marine and ice deposits, respectively (Table 1 and Figs S4–S12). Based on the four established correlations, we predicted the Hg accumulation flux data for each core that does not extend to 2012.

**Table 1.** Database summary and impacts of individual changing factors on the respective natural archive Hg fluxes by using general additive modelling (GAM). Stars represent significant levels of the impacts of variables on the respective natural archive Hg records: three stars represent a level of 0.001, two stars represent a level of 0.01 and one star represents a level of 0.05. A – indicates that the variable does not apply to the respective core type. Numbers in parentheses are F values. A higher F value indicates a higher effect of the variable on the respective natural archive Hg records. For a graphic display, see Figs S7–S14.

		Geographic factors		Environmental factors			Emission-related factors			
Core type	Number of cores in the database	Elevation/ocean depth	Catchment area vs. lake area	Temperature	Precipitation	Greenness fraction	Local anthropogenic emission	Local non-anthropogenic emission	Global total emission	Deviance explained
Lake core	159	*** (36.10)	*** (62.32)	*** (60.74)	*** (5.53)	*** (37.09)	*** (76.10)	*** (37.13)	(0.00)	65.0%
Peat core	28	*** (27.32)	–	*** (16.51)	** (1.02)	*** (16.74)	*** (30.32)	*** (8.13)	*** (5.38)	88.1%
Marine core	25	*** (120.48)	–	*** (15.27)	(0.00)	–	*** (46.63)	(0.70)	(0.00)	85.2%
Ice core	9	*** (6.26)	–	*** (2.98)	(0.00)	–	–	*** (1.82)	** (1.08)	83.0%
Total	221									

Combining the Hg accumulation flux data extracted from literature and the GAM predictions, we compiled a natural archive Hg database from 1700 to 2012 (referred to as ‘the database’). The database showed distinctive patterns of Hg accumulation fluxes in the four types of natural archives over the last three centuries. The averaged Hg fluxes in peat, lake, ice and marine cores—hereafter referred to as ‘peat-Hg fluxes’, ‘lake-Hg fluxes’, ‘ice-Hg fluxes’ and ‘marine-Hg fluxes’—have increased by 5-, 6-, 8- and 9-fold, respectively, culminating in peak contemporary levels at 0.033 ± 0.034, 0.055 ± 0.123, 0.002 ± 0.004 and 0.124 ± 0.175 mg m^−2^ yr^−1^ (mean ± standard deviation; detailed data in Table S3). These substantial variations in changing rates and magnitudes highlight the differences between the Hg deposition mechanisms in the four types of natural deposits and reflect the key responses of those Hg sinks of each ecosystem to the changing atmospheric Hg deposition. Therefore, the following sections further discuss these responses by analysing the deviances and similarities between each type of natural archive Hg accumulation and the respective atmospheric Hg deposition. These responses help in further understanding the changes in Hg accumulation in key regions.

### Responses of lake and peat core records to changing atmospheric deposition

#### Lake-Hg and peat-Hg fluxes generally changed with atmospheric Hg deposition

We compared the natural archive Hg accumulation fluxes with the GEOS-Chem modelled atmospheric Hg deposition at each of the coring locations (Fig. 1b and Fig. S13). The GEOS-Chem model was driven by EDGAR anthropogenic Hg emissions [35] and MERRA2 meteorological data [36]. The modelling generated atmospheric Hg deposition fluxes (total, wet and dry) in 2° × 2.5° grids, which offer a cost-effective balance between simulation accuracy and computational efficiency. Model validation against observations indicated an acceptable error margin of ∼50% (Fig. S13). Our comparison revealed that lake and peat cores exhibited similar figures, with 45% of lake-Hg fluxes and 46% of peat-Hg fluxes between 1980 and 2012 falling within a 1-fold range of their respective modelled total atmospheric deposition fluxes (Fig. S14). However, extremes were noted: 7% of lake-Hg fluxes (11 cores) and 7% of peat-Hg fluxes (2 cores) deviated by >10-fold, surpassing the modelled deposition. Additionally, 55% of lake cores and 48% of peat cores exhibited Hg accumulation trends that aligned with their respective modelled deposition trends over the same period.

Apart from the similarities mentioned above, the key impact factors influencing both lake-Hg and peat-Hg fluxes also showed similar patterns, despite their distinctive deposition mechanisms. We analysed the impact of eight relevant environmental, geographical and emission-related factors on the changes in lake-Hg and peat-Hg fluxes using the GAM approach (see Text S2 for methods). The analysis revealed that local anthropogenic Hg emissions had the most significant impact on both lake-Hg and peat-Hg fluxes, evidenced by the highest F values among all factors, with temperature being the next most influential factor (Table 1). The fact that lake-Hg and peat-Hg fluxes share the same primary and secondary impact factors is likely because lakes and peatlands are both generally covered by stagnant water and these cores were taken from relatively remote areas with limited external disturbances. These conditions make atmospheric deposition the dominant input in these natural archives, making them more susceptible to local atmospheric emissions. The significance of temperature is due to its impact on disrupting the biogeochemical cycles of Hg within lake and peat ecosystems. For instance, rising temperatures can promote vegetation growth and aquatic system productivity. These changes could lead to an increase in vegetation fixation and the input of organic matter-bound Hg into peat and lake sediments [18,37]. Besides, rising temperatures could contribute to glacier retreat, providing additional Hg input from meltwater into proglacial lakes [29,38,39].

The general concordance in trends and magnitudes between the modelled data and lake-Hg and peat-Hg fluxes suggests that both types of natural archives are responsive to changes in atmospheric Hg depositions. Besides, the shared key impact factors—particularly local anthropogenic emissions—indicate that lake and peat cores could be used in tandem to assess the impact of anthropogenic activities, such as the onset of major industries and the implementation of pollution control policies. In the following subsection, we leverage this knowledge to discuss regional changes in lake and peat records.

#### Regional lake and peat records reflected contemporary anthropogenic impact

**Figure 2.**
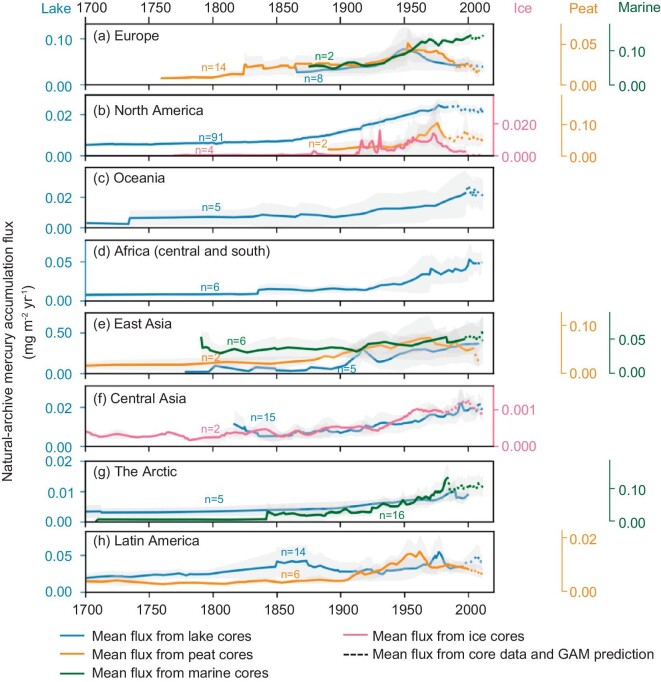
The synthesized regional Hg accumulation fluxes reconstructed from ice, peat, lake sediments and marine sediments from 1700 to 2012. In this context, Africa refers to central and southern areas, Oceania covers Australia and New Zealand, Latin America covers Mexico and the western Andes area, and the Arctic represents Greenland and nearby islands. The shaded areas represent 95% confidence intervals and ‘n’ next to each line indicates the maximum number of cores used in plotting. Here, only the fluxes that were averaged from two or more cores are plotted. Dotted extended lines indicate that the fluxes were calculated by using both core data and predicted values generated by the General Additive Model (GAM). The use of core + GAM predicted data aims to avoid errors induced by inconsistent numbers of cores each year, particularly after 2000, when the number of cores decreased significantly.

From 1900 onwards, lake-Hg and peat-Hg fluxes in all regions started to rise, but their trajectories diverged after the 1950s. Europe was the only region that showed a significant reduction, with lake-Hg and peat-Hg fluxes decreasing by 94% and 97%, respectively, from the respective peaks in the 1950s and 1970s to 2012 (Fig. 2a). In 2012, fluxes dropped to 0.039 (0.026, 0.052) [mean (confidence interval (CI) 2.5%, CI 97.5%)] mg m^−2^ yr^−1^ in lake cores and 0.022 (0.014, 0.030) mg m^−2^ yr^−1^ in peat cores. These 2012 levels closely resemble pre-industrial levels, when the lake-Hg flux was 0.027 (0.015, 0.039) mg m^−2^ yr^−1^ in 1866 and the peat-Hg flux was 0.006 (0.004, 0.008) mg m^−2^ yr^−1^ in 1760 (the earliest year with multiple cores). The significant reductions observed in lake and peat cores in recent decades align well with the decreasing trends and rates of modelled total atmospheric Hg depositions during 1980–2012 (Table 2 and Fig. S15) and with the observed Hg° concentration and Hg^2+^ wet deposition during 1990–2010 [10]. Besides, the reduction rate in lake-Hg (–1.7%/yr) closely mirrored the modelled reduction rates (–1.6%/yr). These general concurrences in decreasing trends and rates between lake and peat cores indicate that similar factors drove these changes. One major driver contributing to these reductions could be the effective implementation of environmental policies aimed at reducing air pollutant emissions from coal burning in Europe. These policies trace back to the UK’s Clean Air Act of 1956, which was prompted by the Great London Smog and has been strengthened by a series of regulations enacted by the European Union since 1970 [40]. Consequently, most European countries have progressively decoupled their economic development from coal consumption (Fig. S16).

**Table 2.** Spatial comparison between natural archive data of Hg accumulation fluxes and modelled total atmospheric Hg deposition fluxes (in brackets) by trend, changing rate and magnitude. Modelled results were extracted from respective coring locations and are presented in brackets for easy comparison. ↑ indicates a general increasing trend, ↓ indicates a general decreasing trend. * indicates the trend is at a significance level of 0.05.

Core type	North America	Europe	Latin America	Oceania	Central and Southern Africa	Central Asia	East Asia	The Arctic
**Trend from 1980 to 2012**								
Lake core	↓ (↑*)	↓*(↓*)	↑*(↑*)	↑*(↑*)	↑*(↑*)	↑*(↑*)	↑*(↑*)	↑*(↑)
Peat core	↓ (↑*)	↓*(↓*)	↓*(↑)				↓*(↑*)	
Ice core	↓ (↑*)					↑(↑*)		
Marine core		↑(↑)					↑(↑*)	↑*(↓)
**Changing rate (%/year) from 1980 to 2012**								
Lake core	–0.3 (0.3)	–1.7 (–1.6)	0.4 (0.6)	1.1 (0.6)	2.2(0.8)	1.3 (0.7)	1.9 (3.7)	2.2 (0.2)
Peat core	–0.2 (0.4)	–0.4 (–0.6)	–0.6 (0.3)				–0.9 (1.8)	
Ice core	–3.5 (0.5)					0.1 (0.7)		
Marine core		0.4 (0.8)					0.6 (1.9)	0.4 (–0.2)
**Mean flux (mg m^−2^ yr^−1^) in 2012**								
Lake core	0.023 (0.014)	0.039 (0.017)	0.034 (0.024)	0.021 (0.018)	0.045 (0.021)	0.020 (0.010)	0.414 (0.017)	0.007 (0.007)
Peat core	0.054 (0.011)	0.022 (0.014)	0.010 (0.017)				0.025 (0.019)	
Ice core	0.0001 (0.006)					0.001 (0.007)		
Marine core		0.144 (0.005)					0.067 (0.006)	0.108 (0.008)
**Maximum number of cores**								
Lake core	91	8	14	5	6	15	5	5
Peat core	2	14	6				2	
Ice core	4					2		
Marine core		2					6	16

**Figure 3.**
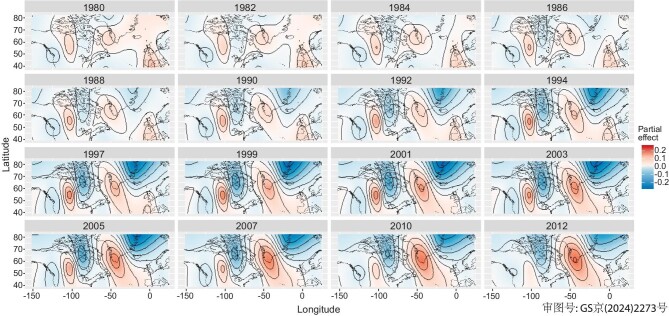
Spatial–temporal variations in natural archive data of Hg accumulation fluxes denoted as partial effects across North America (104 cores with 93% being lake cores), the Arctic (24 cores with 67% being marine cores) and Europe (24 cores with 58% being peat cores and 33% lake cores) from 1980 to 2012, analysed by using GAM. The plots were overlaid with map contours spanning the coordinates 38°N–82°N, 150°W–23°E. The change in partial effects visually demonstrates how Hg accumulation fluxes at specific locations change along with time while holding other variables constant. This spatial–temporal GAM analysis does not distinguish between core types, aiming for a comprehensive comparison among ecosystems and geographical locations. Note that lake and peat cores are more likely to mirror total atmospheric deposition compared with marine cores. Plots show decreasing accumulation fluxes in Europe (terrestrial environment), increasing fluxes in the Arctic region (marine environment) and mixed effects in North America (terrestrial environment).

North America exhibits a mixed flux trend, with lake-Hg fluxes initially increasing at a rate of 1.8% per year until the 1970s, followed by a statistically insignificant decrease (Fig. 2b). By 2012, the lake-Hg flux reached 0.023 (0.021, 0.025) mg m^−2^ yr^−1^, representing a 5-fold enrichment from the pre-industrial level of 0.005 (0.0042, 0.0061) mg m^−2^ yr^−1^ in the year 1700. These overall insignificant decreasing trends in North America resulted from diverse changing patterns of lake-Hg and peat-Hg trends across its subregions. Figure 3 illustrates the spatial–temporal changes in Hg accumulation in North America that were derived from GAM analysis (see Text S2 for methods). The result showed declining Hg accumulation fluxes on the eastern and western sides of North America and increasing Hg accumulation fluxes with slowing year-on-year changing rates in the central region during 1980–2012. These non-uniformed subregional trends from natural archives align with the general decreasing patterns in wet deposition observed in the eastern and western regions of the USA, alongside the increasing [41] or slowing decreasing trends [10] in the central region, depending on targeted periods. The mixed trends are likely a result of locally specific environmental regulations, including the USA’s Clean Air Act of 1970, the continuous reliance on coal in the USA until 2008 (Fig. S16) and contributions from trans-boundary Hg pollution across continents [41].

Unlike the declines observed in Europe and North America, lake-Hg fluxes in Oceania, central and southern Africa and East Asia experienced intensified Hg accumulation up until 2012 (Fig. 2c–e). The recent increases in lake-Hg fluxes agree with the modelled atmospheric deposition during 1980–2012. The increases were likely driven by escalated coal usage in Australia, South Africa and China, which rose by 71%, 190% and 533%, respectively, over the same period [42]. Additional contributions to these increases could have stemmed from Artisanal and Small-Scale Gold Mining (ASGM) activities, which are currently the largest Hg emission source [1]. Triggered in part by surging gold prices after the 2000s, ASGM has proliferated in developing regions worldwide, including central and southern Africa and China [43,44]. However, the magnitude of ASGM emissions carries substantial uncertainties and needs further validation (see Text S4 for more discussion).

In Central Asia, high-altitude lake-Hg fluxes remained constant from the eighteenth century to the 1930s and then increased steadily to 2012, standing at 0.020 (0.013, 0.027) mg m^−2^ yr^−1^ (Fig. 2f). Central Asia is an extensive mountainous region spanning the Third Pole area. Therefore, the region is influenced by global mercury emissions, particularly from neighbouring regions of East Asia and South Asia, both of which have experienced increasing anthropogenic emissions [45]. The high-altitude lake-Hg flux trend aligned with the rising emission trends, as expected. However, the lake-Hg fluxes had a year-on-year changing rate of 1.3%/year during 1980–2012, which surpassed the modelled atmospheric deposition rate of 0.7% at lake locations. This acceleration in the high-altitude lake-Hg changing rate could be attributed to a heightened atmospheric supply of oxidated Hg^2+^ at higher elevations due to the increased availability of oxidants [46–49]. Additionally, high-altitude proglacial lakes also receive Hg inputs from glacier meltwater, which have been enhanced by rising temperatures over recent decades [29,39].

### Responses of marine core records to changing atmospheric deposition

#### Marine cores generally overstated atmospheric Hg deposition

In contrast to fluxes in lake and peat cores, marine-Hg fluxes significantly differed from the modelled atmospheric deposition in trends and displayed significant differences in magnitudes. Notably, 58% of marine-Hg fluxes between 1980 and 2012 showed disparities of >10-fold, with only 19% of marine-Hg fluxes falling within the 1-fold range of the respective total atmospheric Hg deposition fluxes. In general, marine-Hg fluxes were ∼20-fold greater than the modelled deposition (Fig. S14). These substantial differences between marine-Hg fluxes and modelled atmospheric deposition suggest that the factors driving the changes in atmospheric deposition, such as emission control policies, are not the primary drivers of marine-Hg fluxes. This observation is further supported by the GAM impact factor analysis, which revealed that the ocean depth at the sampling location exerted the most significant impact. Ocean depth can alter the marine-Hg fluxes by influencing the physical movement of marine sediments to which Hg binds. These movements include sediment focusing [50] and sediment export to the deep sea [51,52]. Ultimately, the discrepancies in trends and magnitudes between the modelled data and sedimentation records suggest that marine-Hg fluxes are unlikely to respond in a timely or consistent manner to changes in atmospheric deposition.

#### All regional marine cores showed rising trends

Marine-Hg fluxes in the oceans of Europe, the Arctic and East Asia all exhibited increasing trends. These rises are especially noteworthy for Europe and the Arctic, where atmospheric deposition trends were decreasing. In Europe, marine cores showed a significant upward trend of 3% per year before the 1960s, which then slowed to 0.4% per year until 2012. This increasing trend is in stark contrast to the previously discussed declines in the modelled atmospheric deposition of the region and the lake-Hg and peat-Hg fluxes following the pivotal periods of the 1950s and 1970s, respectively. By 2012, marine-Hg fluxes in Europe reached 0.144 (0.140, 0.148) mg/m² per year, ranking among the highest levels within the database.

Similarly, the marine-Hg fluxes in the Arctic showed significant monotonical increasing trends from 1920 to 2012, although the growth rate decreased from 2.8%/year to 0.6%/year after 1980 (Fig. 2g). This contemporary increasing trend contrasted with the decreasing ambient atmospheric Hg concentration of the region, which has declined by –0.95%/year since 1995 based on data from Station Alert at the northern tip of Greenland [7]. The increasing trend also opposed the decreasing modelled atmospheric deposition rate of –0.2%/year during 1980–2012, averaged from marine core locations. By 2012, marine-Hg fluxes in the Arctic stood at 0.108 (0.077, 0.140) mg m^−2^ yr^−1^, designating it as a Hg accumulation hotspot, as shown in Fig. 3.

The sustained rises in marine-Hg fluxes in both the Arctic and Europe could be attributed to various factors. These include the continuous cycling of Hg within the marine environment [53], continuous inputs from coastal erosions [26] and possible enhanced ecosystem productivity in coastal areas [37]. The Arctic region also receives additional Hg inputs from melting Greenland glaciers and permafrost—processes that have been amplified by rising temperatures [54,55]. The increasing marine-Hg flux trends in the Arctic and Europe suggest delayed or limited responses of the marine ecosystems to changing atmospheric Hg deposition. We also acknowledge that the above analyses of marine-Hg fluxes are limited by the smaller number of marine cores (25 cores) compared with peat and lake cores. Therefore, future studies are encouraged to provide additional validation to bolster the findings and further investigate the dynamics of Hg deposition in marine environments.

### Responses of ice-core records to changing atmospheric deposition

#### Ice cores generally under-recorded atmospheric Hg deposition

Similarly to marine cores, Hg fluxes in ice cores also significantly differed from modelled atmospheric deposition in trends and magnitudes. When magnitudes are compared with modelled total atmospheric Hg deposition fluxes, 66% of ice-Hg fluxes from 1980 to 2012 showed disparities of >10-fold, with only 10% within a 1-fold range. In general, ice-Hg fluxes were nine times smaller than the modelled deposition (Fig. S14). The GAM impact factor analysis revealed that the elevation of the sampling location exerted the most significant impact. This finding is likely due to higher elevations correlating with heightened ultraviolet intensity, which could linearly influence the photoreduction process of Hg in ice deposits [56], leading to the loss of deposited Hg. Eventually, all the differences in trends and magnitudes, along with the primary impact factor, suggest that ice-Hg fluxes are unlikely to respond promptly or consistently to changes in atmospheric deposition.

#### High-altitude ice records were prone to natural influences

In the ice-covered mountainous regions of Central Asia, ice-Hg fluxes remained constantly low until the 1930s and then increased rapidly until 1960. Following this period, the fluxes fluctuated at ∼0.001 mg m^−2^ yr^−1^ until 2012. The post-1960 trend of ice-Hg fluxes diverged from the rising trends of both global and regional Hg emissions that affect Central Asia [45]. The fluctuations in ice-Hg fluxes may have resulted from several factors, including the loss of deposited Hg through photoreduction, which is intensified by high ultraviolet radiation at elevated altitudes. Additionally, these fluctuations might have resulted from the release of stored historical Hg as ice melting, driven by rising temperatures, which are particularly pronounced in high-altitude regions [57,58]. Nevertheless, the current analysis is limited by the small number of ice cores available in this region (just two cores). More ice cores are needed to improve the robustness of the trend analysis and provide more convincing evidence.

### Reflections of natural archive responses on global mercury management

#### Revisiting the anthropogenic emissions during the eighteenth and nineteenth centuries

Several global estimates on Hg emissions in the eighteenth and nineteenth centuries revealed an unimodal curve with a peak matching contemporary levels [2,59], largely driven by silver [60], mercury and gold mining [2] during the Spanish colonization (1570–1850) and Gold Rush era (1800 onwards). These high historical estimates were considered overestimated and strongly contested by geochemical records [53,61,62]. Our synthesized regional lake-Hg fluxes in Central Asia and Latin America (Fig. 2h) during the period showed mildly elevated lake-Hg fluxes, supporting the notion that mining emissions were likely overestimated and/or had only local impacts. Consequently, some studies revised these emissions to one-third to half of the original estimates [53,63], using natural archive Hg accumulation fluxes as references. However, it is important to note the influence of sampling locations. If the distances between natural archives and mining locations exceed the local impact range (typically 50 km [64] to 100 km [22]), the natural archive Hg fluxes might be biased when evaluating and calibrating the mining Hg emissions. Notably, no cores from California, USA—the hotbed of the Gold Rush—were analysed by Zhang *et al.* [53] or Engstrom *et al.* [62], nor were any included in this study. An accurate estimate of historical mining Hg emissions is crucial for contemporary Hg management, as legacy Hg pollution can still influence current Hg biogeochemical cycling [63]. Hence, for further revision of the mining emissions, the use of natural archive fluxes for validation or calibration should be approached with caution and additional core samples are needed in key mining areas.

#### Effectiveness of pollution control policies and impact of coal use

The lake-Hg and peat-Hg fluxes in three regional groups (i) Europe, (ii) North America and (iii) East Asia, Africa and Oceania provide a clear basis for comparing the effectiveness of environmental policies. Both Europe and North America pioneered environmental management but exhibited different pollution trajectories. In Europe, contemporary lake-Hg and peat-Hg fluxes had almost returned to pre-industrial levels, indicating effective ecosystem recovery and the success of pollution control measures. In contrast, the declining trends of lake-Hg and peat-Hg fluxes in North America were insignificant. This discrepancy is likely due to the reliance on coal—a major Hg emission source. European countries decoupled their economies from coal use early on, whereas the USA maintained its reliance until 2008 (Fig. S16). A more profound impact of coal use is evident in East Asia, Africa and Oceania, where coal remained a primary energy source at least until 2012. Consequently, lake-Hg fluxes in these regions showed monotonic increases, with East Asia emerging as a particularly intensified Hg accumulation hotspot. The comparison results underscore that general environmental policies can be effective in managing Hg pollution; however, the ongoing use of coal significantly undermines this effectiveness.

#### Critical ecosystems for Hg pollution recovery

Compared with terrestrial lake and peat ecosystems, marine ecosystems are more challenging to recover from Hg contamination. This is evidenced by the substantially reduced contemporary Hg fluxes in lake and peat cores in Europe, whereas marine core fluxes in the same region continued to rise. The difficulties in change partly stem from the fact that marine ecosystems, including ocean water bodies and sediments, represent the largest Hg sink on Earth [1]. Additionally, the open nature of marine systems, with ocean currents mobilizing Hg both horizontally and vertically, significantly diminishes the effectiveness of environmental policies that focus on reducing atmospheric Hg emissions and deposition on marine ecosystem recovery.

High-altitude ecosystems face similar challenges. They are more susceptible to environmental and geographical factors, making recovery difficult through the sole control of anthropogenic Hg emissions. The GAM analysis indicated that elevation is the primary driving factor for changing Hg fluxes in ice cores, and a secondary and tertiary factor in peat and lake cores, respectively (Table 1). At higher elevations, the Hg in natural archives would be affected by the presence of more oxidants [47], greater photochemical reduction [56] and more intense temperature increases [57,58]. Additionally, the dynamic interactions between melting ice and proglacial lakes [29,38,39] under climate change pose new challenges for containing Hg contamination in high-altitude ecosystems.

## CONCLUSION

In summary, we compiled a natural archive Hg record database spanning the years from 1700 to 2012, utilizing data from 221 cores collected from ice, peat, lake and marine deposits across eight key regions. Our analysis focused on how these natural deposits, acting as Hg sinks of respective ecosystems, respond to changes in total atmospheric Hg deposition. Our findings revealed that lake-Hg and peat-Hg fluxes exhibited a strong association with local anthropogenic Hg emissions and mirrored the trend of total atmospheric Hg deposition, albeit with higher magnitudes. This distinct characteristic evidenced the positive effect of past collective environmental policies in Europe in recovering lake and peat ecosystems—at least their sedimentary components—from Hg contamination. Additionally, our study revealed elevated Hg accumulation in lake ecosystems in East Asia, Africa and Oceania, likely driven by economic development, including coal consumption, among other factors. Conversely, ice-Hg and marine-Hg fluxes were primarily regulated by natural processes, such as Hg photoreduction, ice melting and coastal erosions, and thus were not sensitive to changing atmospheric inputs driven by anthropogenic interventions such as emission controls. As a result, we found universal rising trends in marine-Hg fluxes in Europe and the Arctic post the 1950s despite declining atmospheric emissions, concentrations and depositions. Additionally, our findings underscored the challenges in containing Hg contamination in high-altitude ecosystems due to the dynamic Hg exchange and the remobilization of historical Hg through ice melting.

Although natural deposits may not fully represent entire ecosystems, they provide valuable insights into the principal responses from Hg sinks within these ecosystems, which can also act as potential Hg sources. Therefore, we call for targeted mitigation strategies that are tailored to key ecosystems in oceans and high-altitude areas, as well as critical regions such as East Asia and the Arctic. Besides, it is crucial to address Hg pollution and climate change simultaneously [65], as changing natural conditions—such as variations in vegetation types, organism productivity and soil erosion levels—can influence Hg contamination levels and offset the effectiveness of policies. Moreover, there is a need for more paleoenvironmental studies in less-explored natural archive materials, such as ice and marine sediments, and in under-researched but important regions, particularly East Asia, Africa and historical mining regions. These studies are essential for enhancing our understanding of global biogeochemical Hg cycling and supporting assessments of the effectiveness of Hg mitigation policies, especially under the Minamata Convention on Mercury.

To push forward research in this direction, the established natural archive Hg database could potentially help to (i) reconstruct long-timescale, global-gridded, atmospheric Hg depositions, which could be achieved by combining and complementing natural archive Hg records (long temporal scale but limited in special coverage) and global modelled gridded depositions (global coverage but limited in temporal scales)—successful reconstruction can provide valuable Hg data for less-studied areas such as East Asia, Africa and South Asia; (ii) incorporate global Hg cycle modelling to constrain Hg emission estimates, including anthropogenic emissions from sources such as metal mining and aquatic re-emissions; (iii) disentangle climate and socio-economic drivers of Hg accumulation fluxes in the identified key ecosystems, i.e. marine and high-altitude lake systems, and generate more targeted policies and measures for effective ecosystem recovery from Hg contamination.

## METHODS

We conducted this study by following the below processes (for detailed methods, please see Text S2 and Fig. S17):

- Compiled a natural archive Hg flux database by using data from lake, peat, marine and ice deposits.
- Analysed the responses of natural archive Hg fluxes to atmospheric Hg depositions. This step is divided into three parts. First, natural archive Hg fluxes were compared with atmospheric Hg depositions modelled by GEOS-Chem at each coring location to identify disparities and similarities. Second, eight key impact factors that influence changes in natural archive Hg fluxes were examined. Third, spatial–temporal analysis was conducted to understand the responses of natural archives in eight key regions.
- Assessed impacts of anthropogenic activities, including environmental policies and identified critical regions and ecosystems for prioritizing future Hg abatement policies.

This study employed a multidisciplinary approach, incorporating elements from physical geography and atmospheric sciences, which resulted in uncertainties spanning various dimensions. These uncertainties encompass but are not limited to the following aspects (for in-depth discussions, please refer to Text S4):

- Potential bias stemming from core distribution and numbers could be more pronounced in regions such as Southeast Asia, the Arctic, East Asia, central and southern Africa, and South America. Such bias may have affected the accuracy of trend and magnitude analyses.
- Uncertainties in natural archive Hg records arising from different deposition mechanisms. See Table S1 for a summary detailing pre- and post-depositional processes that contribute to differences between natural archive Hg fluxes and atmospheric Hg deposition fluxes.
- Uncertainties in natural archive Hg records arising from chronologies, including dating error ranges and differences in dating methods employed across studies. Consequently, the geochemical Hg records discussed herein should be understood as representing an approximate period of ±10 years, rather than precise years.
- Uncertainties in natural archive Hg records arising from the concentration-to-flux conversion. Contemporary Hg accumulation fluxes in ice and marine cores might have been underestimated due to the consistent sedimentation rates used in the conversion.
- Uncertainties in total atmospheric Hg deposition linked to the transportation and deposition processes modelled in GEOS-Chem. The model employed a coarse resolution of 2° × 2.5°, meaning that the grid-average results might not fully represent Hg deposition at specific core sampling locations. Additionally, the model might have underestimated the total deposition onto ice surfaces and overestimated deposition onto lakes due to uncertainties in high-altitude modelling and lake-Hg re-emissions, respectively. These uncertainties could have affected the accuracy of the magnitude comparisons between modelled and natural archive Hg fluxes.
- Uncertainties in Hg emission estimates on ASGM, which may have further led to overestimation of the GEOS-Chem-modelled atmospheric deposition in East Asia, central and southern Africa, and Latin America in 1980–2012.

## Supplementary Material

nwae417_Supplemental_Files

## Data Availability

The natural archive Hg flux data and core information, including references, of the selected 221 cores are available in Dataset S1. Ground observation data of wet mercury deposition and concentrations are available in Dataset S2. The data used to plot Figs 1–3 are provided in Dataset S3. The GAM codes are available upon reasonable request to the lead contact, Shuxiao Wang (shxwang@tsinghua.edu.cn).
