# Supplementary material for: Mercury records from natural archives reveal ecosystem responses to changing atmospheric deposition
Source: Natl Sci Rev. 2024 Nov 19;11(12):nwae417. doi: 10.1093/nsr/nwae417 (PMC11660912; doi:10.1093/nsr/nwae417)
Supplement: nwae417_Supplemental_Files [file nwae417_supplemental_files.zip › NSR Natural archive Hg - SI - final.docx]

**Supporting Information for**

**Mercury records from natural archives reveal ecosystem responses to changing atmospheric deposition**

**This PDF file includes:**

Text 1 to 4

Figures S1 to S17

Tables S1 to S5

SI References

**Other supporting materials for this manuscript include the following:**

Datasets S1 to S3

**Supporting information Text**

# Comparative analysis between this study and other mercury pollution-related studies using natural archives

Several studies have examined mercury pollution using natural archives, encompassing global-scale reviews[1-3] and regional analyses such as those in the Americas[4] and the Arctic[5]. These studies address various topics, including understanding deposition mechanisms in natural archives, validating emissions from Artisanal and Small-Scale Gold Mining (ASGM), and comparing Hg accumulation levels between the northern and southern hemispheres. These studies share certain common traits. Firstly, they primarily offer qualitative analyses of impact factors. Secondly, the regional trends, if any, are typically illustrated by stacking plots of individual cores, making it challenging to generate general trends. Lastly, data comparisons often rely on trends and qualitative assessments.

This study builds upon previous reviews in several ways. Firstly, it employs quantitative analysis of impact factors using Generalized Additive Models (GAM). Secondly, it compares synthesised regional fluxes based on both trend and magnitude. This regional comparison is facilitated by specific processes, including careful core selection to ensure a similar base for reflecting atmospheric deposition, temporal interpolation for obtaining annual Hg fluxes, and GAM prediction to fill data gaps between 1980-2012 in individual cores.

Furthermore, this study comprehensively compared individual core data with the respective GEOS-Chem modelled total atmospheric mercury deposition from 1980 to 2012, examining trends, changing rates, and magnitudes. These comparisons provide insights into how natural archives respond to changing atmospheric total deposition, highlighting disparities between natural archive data and atmospheric deposition. Caution is thus advised in future studies when making inferences about atmospheric deposition and conducting comparisons with atmospheric emissions using natural archive data.

Lastly, despite serving different research purposes, our study collected and analysed a larger number of cores (221) compared to other reviews. The compiled raw flux data from individual cores and the synthesised regional flux data are publicly available (Dataset S1 and S3), offering valuable resources for advancing future research in the field.

# Methods

## Database compilation of natural archive mercury records

*Core selection for the database.* To compile the Hg accumulation flux database, we focused on peer-reviewed journal papers published before August 2022 using the search engine Web of Science. The search was done by all keyword combinations between the pollutant (‘mercury’ OR ‘Hg’) and natural archives (‘lake sediment’ OR ‘marine sediment’ OR ‘lake’ OR ‘peat’ OR ‘peatland’ OR ‘sediment’ OR ‘ice’ OR ‘glacier’ OR ‘snow’ OR ‘soil’). We carefully selected papers and cores based on the following criteria:

1. The Hg preserved in cores was primarily sourced from atmospheric deposition. For instance, (a) we excluded cores from small lakes that receive sewage discharges, (b) excluded marine cores at estuaries directly receiving terrestrial river inputs, (c) preferably chose hydrologically closed lakes, and (d) preferably chose ombrotrophic (rainfed, thus only atmospheric input) peat cores or minerogenic peat with ombrotrophic characteristics than other kinds of peat cores.
2. Coring places were not disrupted by significant human activities, e.g., sediment cleaning and peat burning, or affected by natural geophysical processes, e.g., earthquakes, landslides, and hydrodynamic mixing of sediments.
3. Cores have no significant post-depositional movements, including diagenesis processes, as indicated in the papers. Note that this criterion relies solely on the assessment of the paper authors, which could contain errors. Furthermore, the absence of any mention of post-depositional movements in the papers does not necessarily imply the absence of such movements.
4. The disclosed Hg data include accumulation flux (mg/m^2^/year) or the respective papers disclosed Hg concentration (mg/kg) together with other necessary archival information that enables the conversion from Hg concentrations to Hg fluxes.
5. The Hg fluxes were determined in a temporal resolution finer than 20 years, which is the approximated range of dating errors of lead-210[6] and carbon-14[7] in materials with ages ≤300 years, to avoid large chronological errors induced by dating techniques.

*Data acquisition and compilation*. After the selection, we focused on 221 cores and extracted the following data and information: (a) Hg-related data, including Hg flux and Hg concentration, (b) physical properties of cores, including chronology, sedimentation rate, and density, (c) environmental settings, including core coordinates, elevation, region and country, surrounding land cover, nearby Hg emission sources, and possible disturbances. (d) In the case of lake cores, we also collected data on catchment area, lake area and sediment-focusing factor if provided.

We obtained Hg flux data from (a) authors through email requests, (b) tables or SI database if provided in the papers, and (c) figures using WebPlotDigitizer (https://apps.automeris.io/wpd/), a free online tool for data extraction from figures, and (d) converted from concentration using eq1. Specifically, the concentration-to-flux conversion was applied to a total of 36 cores (16% of the total cores), they are three ice/snow cores, four peat cores, 10 lake cores, and 19 marine cores. To obtain the closest estimates of Hg fluxes from conversion (eq 1), we calculated sediment accumulation rates (kg-sed/m^2^/yr) over different sedimentary sections if the papers provided with section parameters including core depths (m), age spans (age_section-top_ – age_section-bottom_), and sediment densities (kg-sed/m^3^). Otherwise, we adopted the disclosed full-core-averaged sedimentary accumulation rate to perform the conversion, which was mostly in marine cores.

$Hg Flux =Hg Concentration \times sediment accumulation rate$

eq 1

With the raw Hg flux data prepared, we used linear interpolation to fill gaps between years to obtain annual Hg flux data. The annual Hg accumulation flux data and respective core information were compiled into the Hg accumulation database (Dataset S1).

*Database cross-check.* We also conducted two rounds of data cross-checks. First, the data in the database were compared with data in respective papers to eliminate errors from manual data extraction. Second, we compared the flux data within the database to check the consistencies of core data in the same region, the same type of natural archive, and the same coring location to find erroneous values from published papers, which may be derived from human errors, including wrong unit used and wrong data disclosed. If found inconsistency, we contacted the corresponding authors of the papers to validate the published data. If we received authors’ updates, we replaced the wrong data with the updated one in our database; if no response was received as some papers were published decades ago, we removed the data from our database. Eventually, we gathered and compiled Hg deposition flux data from 221 cores in 70 papers, covering eight regions and 34 countries and subregions from 1700 to 2012.

## GEOS-Chem modelling of global total atmospheric Hg depositions

We employed the Global Earth Observing System Chemistry model (GEOS-Chem, version 12.6.3, http://geos-chem.org) to generate global total atmospheric Hg deposition fluxes (90°S–90°N, 180°W–180°E, resolution 2°×2.5°) during 1980-2012. The GEOS-Chem model was driven by MERRA2 meteorological data (2°×2.5°) [8], anthropogenic atmospheric mercury emission (0.1°×0.1°), biomass burning emissions (0.25°×0.25°, https://www.globalfiredata.org/), and internal calculation of ocean flux, soil emissions and re-emission[9]. All the input data were calculated at the resolution of 2°×2.5°. Within the framework of GEOS-Chem, the depositions of three distinct Hg species were simulated: gaseous elemental Hg (Hg^0^), gaseous oxidized Hg (Hg^2+^), and particle-bound Hg (Hg^P^), encompassing both dry and wet deposition processes. The modelled atmospheric Hg deposition fluxes referenced in this article are the collective Hg deposition, representing the cumulative sum of the three aforementioned forms of Hg species.

The GEOS-Chem simulation of Hg consisted of wet and dry deposition[10]. Wet deposition exists in Hg^2+^ and HgP by means including washout from precipitation (rain and snow) and scavenging in convective updrafts[11]. The modelled ambient Hg concentrations and wet deposition fluxes were validated using ground observations obtained from the Atmospheric Mercury Network (AMNet, <https://nadp.slh.wisc.edu/networks/atmospheric-mercury-network/>), Mercury Deposition Network (MDN, <https://nadp.slh.wisc.edu/networks/mercury-deposition-network/>), the Global Mercury Observation System (GMOS)[12] and observations reported in published journal articles (SI Dataset S2). Normalized mean error (eq 2) was adopted for the evaluation of modelling accuracy.

$Normalized mean error=\frac{\sum_{i} |Simulation_{i}-{Observation}_{i}|}{\sum_{i} {Observation}_{i}}$

eq 2

Dry deposition refers to the Hg removal process by turbulent transfer and uptake at the ground surface and is observed in Hg^2+^, HgP, and Hg^0^. The deposition speed and flux of dry deposition were modelled with a standard resistance-in-series scheme[11, 13, 14], including aerodynamic resistance, atmospheric boundary layer resistance, leaf stomatal and cuticle resistance, and soil resistance[13, 15, 16]. Dry deposition flux highly depends on elevation, atmospheric mercury concentrations, ground surface conditions, and meteorological conditions[17-19]. Compared with wet deposition, it is more difficult to validate the modelled dry deposition with ground observations because measuring methods are not standard and consistent[20-22].

The validation of modelled wet deposition and atmospheric concentration of Hg showed normalized mean errors of 51.4% and 16.7%, respectively (SI Fig. S4). Both errors fall within an acceptable range, consistent with other studies that reported wet deposition estimates with mean errors around 50%, with higher errors in high-altitude/latitude areas[23-25]. However, validating the modelled dry deposition is more challenging due to the lack of standardized ground observation methods[20]. See SI Supporting Text 4 for an extensive discourse on modelling result uncertainty. In the absence of more precise alternatives, we prudently adopted the modelled total (wet + dry) deposition fluxes as a surrogate for actual atmospheric deposition fluxes.

## General Additive Model (GAM) analysis

For cores with upper temporal limits of chronology younger than 2012, we extended the respective Hg accumulation data to 2012 using GAM prediction. The model GAM is powerful in prediction based on a relationship between the response variable with the transformed smooth functions of independent variables[26]. We conducted a non-linear correlation analysis on the impacts of environmental, geographic and emission-related factors on Hg fluxes with each of the four types of natural archives using the gam package in R. The GAM analysis was conducted for each type of natural archive following procedures (a) variable selections, (b) core selections, (c) GAM model fitting, and (d) validations and diagnostic check.

*Variable selection.* We collected variable data from global open-source databases and peer-reviewed papers compiled in the database (SI Table S4) in 1980-2012, a period overlapped by MERRA-2 [8], EDGAR [27], and the database. A total of eight variables, out of 20, were selected based on two considerations. First, variables should be independent of each other. The inclusion of a variable should not lead to significant multicollinearity between variables, as indicated by Variance Inflation Factors of all variables should be smaller than 5 and as close to 1 (no correlation) as possible[28, 29]. Second, the inclusion of a variable should improve the overall goodness of fit of the GAM model, indicated by a reduced Akaike information criterion value.

*Core selection for GAM analysis*. While the cores in the database were meticulously chosen, they might still contain errors, stemming from the original papers or our selection process. These errors could become apparent when comparing natural archive Hg records to modelled atmospheric total deposition fluxes. To ensure the precision of the input data for the GAM analysis, we excluded cores that meet the following criteria:

1. peat and lake cores whose accumulation fluxes deviated by more than 10-fold from the modelled atmospheric depositions. Such discrepancies may result from either unaccounted deposition mechanisms that were irrelevant to atmospheric depositions in respective papers or uncertainties of modelled atmospheric depositions.
2. cores with likely inaccurately estimated emissions in the EDGAR dataset in the corresponding grids.

All the excluded cores have been noted in Dataset S1 along with their exclusion reasons.

*GAM model fitting.* We formulated GAM models for each of the four types of natural archives as eq 3-6 and fitted them using Gamma error distribution with a log link, which is suitable for positive-only data[30]. Smooth function (s) of each variable were configured with a default number of smooth functions (k = 10), a smooth class of a cubic spline basis (bs = ‘cr’), and the “REML” method. We trained the models with natural archive flux data from 193 cores and the variable data at respective coring locations between 1980-2012. The variables in brackets presented below are self-explanatory, see SI Table S4 for a detailed explanation of individual variables. The GAM models generate the partial effect of each of the predictor variables on the response variable while holding all other predictor variables constant. Partial effect measures the relationship between the predictor and the response, accounting for the potential nonlinearities and interactions with other variables. A partial effect of 0 suggests that there is no association between the predictor and the response. Values larger/smaller than 0 indicate increasing positive/negative effects.

$Hg\_Ice\sim s\left( Elevation \right)+ s\left( Precipitation \right)+ s\left( Temperature \right)+s\left( Greenness \right)+ s(Global\_TotEmit) + s(Local\_AntEmit)+ s(Local\_nonAntEmit)$

eq 3

$Hg\_LakeSed \sim s(Elevation ) + s(Precipitation) + s(Temperature ) + s(Greenness) + s(Global\_TotEmit) + s(Local\_AntEmit) + s(Local\_nonAntEmit ) + s(CatchmentArea/LakeArea)$

eq 4

$Hg\_Peat\sim s(Elevation) + s(Precipitation) + s(Temperature) + s(Greenness) + s(Global\_TotEmit) + s(Local\_AntEmit) + s(Local\_nonAntEmit)$

eq 5

$Hg\_MarineSed\sim s(Depth)+ s\left( Precipitation \right)+ s\left( Temperature \right) + s(Greenness) + s(Global\_TotEmit) + s(Local\_AntEmit)+ s(Local\_nonAntEmit)$

eq 6

*GAM model* *validation*. The GAM models were validated by checking 1) if the explained deviance is at an acceptable level (normally > 50%); 2) if model fitted value versus core values are close to 1; 3) if the histogram of residuals is close to normal distribution; and 4) if a Q-Q plot with quantiles points closely lie on the 1:1 reference line. If passed validation, the model generated partial effects of each of the eight variables on the respective natural archive Hg records were accepted.

The GAM models were also subjected to 10-fold cross-validation, and the results were presented in SI Table S5. In 10-fold cross-validation, each dataset was randomly divided into ten equally sized subsets (folds). Each model was trained on nine of the folds and validated on the remaining fold. This process is repeated ten times, each time using a different fold as the validation set [31]. The performance metrics from each iteration were averaged to provide an overall evaluation of the model’s performance, indicated by the scale parameter of the GAM model (GAMscale) and Mean Squared Error (CV-mse-GAM). GAMscale represents the dispersion or variability of the residuals (errors) in the model, thus the lower the GAMscale value the better. Similarly, a lower CV-mse-GAM value indicates the model’s prediction is closer to the true values on average during cross-validation. Especially, the same value of GAMscale and CV-mse-GAM indicates the GAM model is well-fitted with an appropriate level of complexity to capture the relationships between predictors and the outcome variable[32].

*GAM prediction.* We fed the satisfied GAM models (eq 3-6) with the eight independent variable datasets at coring locations spanning the period 1980-2012, and the models thus provided predicted Hg fluxes over the same period. Based on the available core Hg flux data in each core, we quantified the deviation of the respective predicted Hg fluxes. Only when the mean deviance of the predicted fluxes fell within the one-fold range of the core fluxes, the predicted flux data would be allocated to the years of missing data for that core. In cases where the conditions were not met, the predicted values were considered unreliable, and the core fluxes were thus not extended.

*GAM spatial-temporal analysis.* We also conducted spatial-temporal GAM analysis by analysing partial effects of year and location on natural archive Hg records worldwide (221 cores) and regional scale covering Europe, North America and the Arctic (152 cores) during 1980-2012. The spatial-temporal GAM analysis used a smooth term (*f*) with a tensor product of the year and coordinates (eq 7)[33].

$Hg\_CoreFluxes \sim f(Year, latitude, longitude)$

eq 7

The GAM result of spatial-temporal analysis for changes in natural archive Hg records was validated by comparing the characterized trends from the regional plots with relevant trends from published independent research. The validation was also performed by comparing the partial effect results in the regional plots with those in global plots (SI Fig. S17). An agreement also indicates good stability and representation of the spatial-temporal analysis.

## Synthesised regional fluxes

The regional Hg fluxes were computed by averaging the Hg flux data, either natural archive records or modelled total atmospheric deposition, in the same region each year. The respective 95% confidence intervals were also determined. For the natural archive Hg records, the synthesised regional Hg accumulation fluxes during 1700-1980 were calculated solely using data from cores. For the fluxes during 1980-2012, both core data and GAM predictions were used to estimate the mean flux and confidence intervals. The inclusion of GAM predictions is because the number of available cores post-1980 reduced significantly, which may lead to wrong estimates and erroneous conclusions.

## Disparities in magnitude, trends, and changing rate between natural archive Hg records and modelled atmospheric deposition

We quantified the disparities ($\delta$) between core-based natural archive Hg records ($f_{core}$) and the modelled total atmospheric Hg deposition fluxes in the corresponding grids of the coring locations ($f_{modeled}$) based on eq 8.

$\delta=\frac{(f_{core}-f_{modeled})}{f_{modeled}}\times100\%$

eq 8

The changing rate and trend were determined based on linear regression of data points over the selected periods. The significance of the trend was estimated by Mann-Kendall Test, which is a statistical assessment of existing monotonic upward or downward trends[34], using the Kendall package in R. We used a two-sided p-value lower than 0.05 to reject null hypotheses of no monotonic trend.

# Natural and anthropogenic impacts on mercury accumulation in natural archives

## Emission-related impacts

Local anthropogenic emissions exerted the foremost influence on lake-Hg and peat-Hg fluxes, evident from the highest F value, indicating the weight of an impact factor. The emissions also significantly influenced marine-Hg fluxes, indicated by the second-highest F value. The implications of changing local anthropogenic emissions on individual sedimentary Hg fluxes were depicted in the partial effect plots presented in SI Fig. S7-14. The partial effect shows the relative change in sedimentary Hg flux levels with variations in the targeted impact factor while keeping other impact factors constant (*ceteris paribus*). These plots show general positive partial effects of local anthropogenic emissions on peat-Hg, lake-Hg, and marine-Hg fluxes, implying that higher local anthropogenic emissions corresponded to increased Hg accumulation fluxes in peat, lake, and marine sediments.

Additionally, local non-anthropogenic emissions exhibited a moderate yet statistically significant influence on lake-Hg, peat-Hg, and ice-Hg fluxes. However, the nature of these partial impacts could be either positive or negative, lacking a consistent directional pattern (see SI Fig. S7,9,11). This fluctuation is likely attributed to the diverse composition of non-anthropogenic emissions, including direct emissions and re-emissions stemming from soil, vegetation, geogenic activities, and biomass-burning activities.

Global total emissions can also significantly affect ice-Hg and peat-Hg fluxes. Notably, after 1990, when global Hg total emissions exceeded 6000 tons/year, the higher emissions led to lower peat-Hg fluxes (SI Fig. 7). However, this negative correlation is an artefact resulting from uneven distribution of peat cores worldwide. A substantial 56% of peat cores in the database were in Europe, a region with decreasing total Hg emissions since the 1950s[35, 36]; the increasing global emissions were contributed from other regions, e.g., in East Asia, which potentially have limited direct impacts on peat-Hg fluxes in Europe. This disparity emphasized the importance of carefully considering core distribution when interpreting sedimentary Hg fluxes. Therefore, we confine the discussion of peat-Hg fluxes only in Europe in the upcoming section of spatial-temporal analysis to avoid uncertainties introduced by uneven core distribution.

## Surface temperature impacts

The annual average surface temperatures recorded at the coring locations spanned a range from -54°C to 28°C. SI Fig. S11 shows an increasing positive partial effect, i.e., higher ice-Hg fluxes, as temperatures decreased from -20°C downwards. This increase in fluxes under colder conditions could be attributed to the heightened ice stability because lower temperatures contribute to reduced sublimation[37] and a decreased likelihood of melting during summertime, thereby minimizing the loss of accumulated Hg. Furthermore, at lower temperatures, Hg may be “cold-trapped” and accumulated due to elevated levels of Hg condensation and fractionation from the atmosphere[38].

For marine cores, the GAM result generally shows a negative correlation between temperature and marine-Hg fluxes. Notably, ice melting in the marine environment might have great impacts on marine-Hg fluxes, as eliminates physical barriers and enables the evasion of water-saturated Hg from oceans[39]. Multiple factors in the coastal environment could change marine-Hg fluxes. For instance, 1) melting glaciers and permafrost, as in Greenland[40, 41], could transport sediment-bound Hg to continental shelves[42]. 2) Elevated temperatures may modify coastal land cover and increase wildfires[43], enhancing natural Hg emissions and atmospheric deposition. 3) Rising temperatures may increase coastal ecosystem productivity[44], providing greater input of organic matter that bounds Hg[45].

In the terrestrial environment, we found a general positive lake-temperature correlation when temperature above 0 °C, which is likely because warmer temperatures enable the growth of broadleaf plants that actively uptake ambient Hg[46] in catchments, amplifying organic matter-bound Hg supply[45]. Such vegetation impact was measured in GAM by greenness fraction, revealing the third most substantial impact on lake-Hg fluxes following temperature as the second. Moreover, the vegetation impact on lake-Hg fluxes could be further heightened by larger catchments[47, 48], a pattern supported by GAM result demonstrating elevated lake-Hg fluxes with larger catchment-to-lake area ratios (≥ 25) (SI Fig. S9). Akin to marine-Hg fluxes, elevated temperatures could stimulate lake primary production, contributing organic matter-bound Hg to lake sediments[49, 50]; however, such a relationship is not universally observed[5, 51]. Last but not least, meltwater from retreated glaciers has introduced additional Hg inputs aside from atmospheric deposition into the proglacial lakes, mostly in high mountain and polar regions[52-54].

The positive correlation between peat-Hg fluxes and temperatures > ca. 7°C is likely a result of a heightened organic matter-bound Hg input from vegetation growth similar to that happened in lake-Hg fluxes, which outran the simultaneously enhanced microbial activity that decomposes peat[55, 56]. Hence, more Hg was retained. Besides, elevated temperatures potentially shorten the duration of the annual freezing period and the depth of frozen peat. Consequently, a greater proportion of Hg is retained within the peat, rather than being expelled alongside pore water during frost exclusion[57]. Moreover, the elevated temperatures are likely to diminish the surface humidity levels of peat, thereby suppressing the generation of water-soluble methylmercury. Therefore, more Hg was retained within the peat, as opposed to its depletion through outflows as methylmercury[58, 59].

## Depth/elevation impacts

The bathymetric depths or topographic elevations at coring locations ranged from -960 masl to 5750 masl, and emerged as a paramount factor influencing ice-Hg and marine-Hg fluxes, while also significantly impacting peat-Hg and lake-Hg fluxes (SI Fig. S7 and S9). For bathymetric depths of marine cores, a positive correlation was observed at depths from -100 masl to 0 masl, i.e., the shallower depths, the higher marine-Hg fluxes, possibly indicative of coastal non-atmospheric inputs such as shore area erosion. Conversely, a negative correlation, i.e., the deeper depths, the higher marine-Hg fluxes, were observed at depths below -300 masl, which may result from sediment focusing[60] and/or sediment export to the deep sea[61, 62]. Besides, the ocean serves as a reservoir of historic anthropogenic Hg emissions, with two-thirds stored in water shallower than -1000 masl[63], and Hg concentrations in ocean water columns generally increase with depth, although not always monotonically[62, 63]. Such elevated Hg inventories and concentrations at deeper areas of coastal regions could also increase marine-Hg fluxes at deeper depths.

At higher altitudes, topographic elevations influence Hg accumulation in natural archives not only through inherent deposition mechanisms but also by the supply of atmospheric Hg deposition. Primarily, reduced availability of total Hg deposition at high-altitude natural archives might arise due to a significant drop in ambient Hg concentrations beyond the planetary boundary layer at moderate altitudes of 2000-3000 masl[64]. However, a heightened supply of water-soluble Hg^2+^ could be anticipated at high altitudes, converted from Hg^0^ owing to increased available free radicals and oxidants, such as bromine and ozone[38, 65, 66]. Moreover, high-elevation areas of the Tibetan Plateau were found with a gradually amplified warming peaking at around 5000 masl due to climate change[67, 68]. This warming may affect land covers especially the ice/snow-covered areas[69, 70] potentially altering Hg supplies to lake cores. As an amalgamated effect, we found a generally positive partial effect of elevations on ice-Hg fluxes, but this positive partial effect gradually diminished beyond ca. 4000 masl. Concurrently, a reducing negative partial effect of elevations on lake-Hg fluxes was also observed beyond this threshold. Eventually, the negative partial effect of elevations on lake-Hg fluxes gradually transitioned to a positive partial effect at higher elevations > 4000 masl. These relationships underscore the plausible risks of Hg pollution in high-altitude ecosystems.

# Uncertainty analyses

## Bias induced by core count and distribution

We acknowledge that this study is subject to unavoidable limitations, primarily stemming from insufficient natural archive records in certain regions. Cores extracted from East Asia, Southeast Asia, South America, and Antarctica are notably scarce. Synthesised regional fluxes derived from fewer cores (< 5) typically exhibit higher uncertainties, as reflected in generally wider confidence intervals in manuscript Figure 3. Additionally, the distances between core locations and emission sources significantly influence natural archive records. For instance, East Asia, with only five lake cores and two peat cores, displays conflicting trends after 1970. This discrepancy arises because the peat cores are not in the same region as the lake cores, and the low number of peat cores compromises the reliability of trends representing peat contamination levels in East Asia. In fact, East Asia shows increasing anthropogenic Hg emissions in EDGAR and elevated atmospheric Hg depositions in GEOS-Chem models from 1980 to 2012 (refer to SI Fig. S15), likely to enhance the Hg accumulations in lake and peat ecosystems.

To mitigate the chances of misinterpretation, our manuscript primarily discusses synthesised fluxes derived from relatively larger numbers of cores (≥ 5) and those derived from fewer cores but with narrow confidence intervals. Due to these considerations, we refrain from presenting synthesised results for Southeast Asia and Antarctica (both having only two cores with large confidence intervals). Nevertheless, the raw flux data of individual cores and synthesised regional fluxes are available in Dataset S1 and S3.

Moreover, no cores were extracted from key countries such as India, Indonesia, and Brazil, which are significant global Hg emission sources, particularly from coal burning and/or artisanal and small-scale gold mining (ASGM)[71, 72]. Therefore, more targeted paleo studies are needed to address these data gaps in the future. Such studies would be valuable for understanding pollution status and facilitating the evaluation of policy effectiveness. The representativeness of the synthesised regional Hg fluxes in the aforementioned areas requires further validation and updates with new data in future studies.

## Uncertainties induced by deposition mechanisms of natural archives

The four types of natural archives exhibit distinct physiochemical mechanisms that can lead to the loss or aggregation of Hg within these archives. We have summarized the impact factors contributing to differences between natural archive Hg records and atmospheric Hg deposition, categorizing these impacts into two phases: before (pre-deposition) and after (post-deposition) Hg reaches and is preserved in natural archives (details see SI Table S1 below). For more comprehensive reviews, see[1, 5, 73].

As a result, not all natural archive data are comparable or suitable for inferring atmospheric deposition. To enable such comparisons, we conducted careful core selection, focusing on cores proven to be primarily affected by atmospheric deposition (refer to the SI supporting Text 2). Additionally, we further avoided dilution or enrichment of Hg in natural archives due to varying sediment densities and accumulation rates by using data of flux (mg-Hg/m^2^/yr) instead of concentration (mg-Hg/kg-sediment).

Certain factors remained unavoidable by simple means of core selection or concentration-to-flux conversion; hence, they were addressed either quantitatively (utilizing GAM results) or qualitatively and discussed in detail in the main text. Note that the same impact factor might have varying effects on different cores of the same type, and each core possesses unique conditions and is subject to different kinds of impact factors.

## Uncertainties induced by chronologies of natural archives

This study analysed 221 cores from 70 individual papers, with the earliest publication dating back to 1989. Given the extensive number of sources, spanning several decades, cross-checking or validating chronological data proved challenging, unlike the scrutiny applied to flux data, where magnitudes were compared among cores from the same regions or coring locations.

In reality, all the determined chronologies bear uncertainties, and sometimes errors, and the dating methods adopted across studies were not uniform. Different methods can yield chronologies with variations of up to 100 years[74, 75]. Discrepancies may arise from various factors, including sample collection and handling[73], dating techniques (such as ^210^Pb, ^14^C, and counting stratigraphical layers), calibration with independent information (such as nuclear fallout of ^137^Cs, ^226^Ra, ^3^H and ^239^Pu, tephra layers, and nearby tree-rings). Besides, in multi-core studies, only a limited number of primary cores are dated using instruments, while others are dated using wiggle-matching based on tie points like flux peaks, introducing potentially larger uncertainties.

Furthermore, for this study, linear interpolation was employed between dated sediment layers to obtain annual data. Despite the effort of core selection to constrain the temporal resolution of cores to be finer than 20 years, which is the approximated range of dating errors for ^210^Pb ^[6]^ and ^14^C^[7]^, such linear interpolation may still introduce chronological errors. Therefore, the natural-archive fluxes discussed in this study are not tied to an exact year but rather to a period of approximately ±10 years.

## Uncertainties induced by concentration-to-flux conversion

Sixteen per cent of the total cores, primarily ice and marine cores, underwent concentration-to-flux conversion using constant sedimentation rates. However, sedimentation rates are seldom constant over time, and typically, more recent periods exhibit higher sedimentation rates than older ones. Consequently, the Hg accumulation fluxes in modern times may be underestimated, and those in the pre-industrial period may be overestimated. Therefore, the flux results should be considered as a lower (or upper) bound.

## Uncertainties of modelled fluxes of total atmospheric Hg depositions

As mentioned in the manuscript, the modelling validation results showed that the normalized mean errors of the modelled wet deposition and modelled atmospheric Hg concentration were 51.4% and 16.7%, respectively (SI Fig. S4); both are at acceptable levels. The modelled wet deposition result shows a general overestimation (underestimation) below (above) ca. 4 µg/m^2^/yr. Compared with wet deposition, it is more difficult to validate the modelled dry deposition with ground observations because measuring methods are not standard and consistent^[20]^.

The accuracy of modelled atmospheric Hg deposition fluxes could be affected by the following factors including but not limited to 1) under/overestimated emissions in EDGAR, which could affect the accuracy of the modelled deposition fluxes. EDGAR adopted a top-down approach that assigns national emissions to 0.1°×0.1° grid based on proxies like populations and roads[76]. 2) Modelled deposition fluxes at high altitudes may entail larger uncertainties than near ground due to errors in air convection modelling[23]. 3) A total of 20%-40% of atmospheric Hg inputs to lake surfaces may be lost due to evasion as a result of photochemical and biological reductions of water-soluble Hg^2+^ to gaseous Hg^0^[77, 78]. However, the varying evasion rates were set constant at 20% and lake emissions were not differentiated from river emissions in GEOS-Chem[23], which may overestimate the modelled fluxes. 4) There was unavoidably information loss due to grid averaging in GEOS-chem modelling (2°×2.5°).

The uncertainties in modelling wet and dry deposition could have had an influence on accuracy when utilized as a benchmark for comparison with natural-archive fluxes. However, this impact is likely limited to magnitude comparisons rather than trends or changing rates. Besides, the subsequent impact factor analysis using GAM and spatial-temporal analysis employing synthesised natural-archive Hg fluxes were both conducted independently of the modelling outcomes and thus are free from the modelling uncertainties.

## Uncertainties of atmospheric mercury emissions from metal mining

Artisanal and small-scale gold mining (ASGM) retains the use of Hg to extract gold, was believed to top coal combustion and contributed the highest amount of atmospheric Hg emissions (838 tons) in 2015, albeit with large uncertainties (675-1,000 tons)[35, 36]. Interestingly, the gold production in South Africa, which used to be the world’s largest gold producer, declined from ca. 700 tons/year in 1980 to 121 tons/year in 2017 (SI Fig. S16) with 9.4% contributed by ASGM [72]. Such large gold production was partly contributed by ASGM, either regulated or illegally. South Africa was among the largest exporters of illegally sourced gold in Africa [79, 80], although there is no official data disclosing ASGM's contribution to total gold production. If assuming the ASGM gold production changes in parallel with the nation’s total gold production, the respective ASGM Hg emissions in South Africa theoretically should also decline no matter estimated with a constant emission factor of losing 75% Hg-used[81] or considering more compliance with environmental regulations that lower the emissions over time. The declining emission trend, however, contradicts the continuous rising accumulation trend revealed by a nearby lake core located in Lesotho[82] (SI Fig. S16), which is under the impact of both coal-fired power plants (the nearest is 200 km away, SI Fig. S17) and ASGM emissions (coring site is inside of the nearest ca. 100 km^2^ grid of modelled emissions[81]) in South Africa. Therefore, the demise of the former world’s largest gold producer seemed to have little impact on the Hg accumulation ca. 100 km away, which implies a possible overestimation of ASGM emissions and/or such emissions might only be of local impact and a likely more significant impact of coal combustion. More follow-up studies are needed to further validate and eventually narrow the uncertainties of ASGM emissions.

**Table S1** A summary of pre- and post-depositional processes of Hg to natural archives contributing to differences between natural archive Hg records and atmospheric Hg deposition. Those process impacts were either avoided, analysed or remained undealt. The treatment of each process was indicated by respective numbers listed in the respective columns.

| Natural deposit | Processes with impacts | Impact avoided by core selection | Impact avoided by flux conversion | Impact avoided by adjustment (done by original papers) | Impact analysed in GAM | Remaining impacts |
| --- | --- | --- | --- | --- | --- | --- |
| Ice/snow | **Pre-deposition**   1. Hg photochemical reduction (depletion)[83]. 2. Recovery of depleted Hg on ice surface due to sea-ice dynamics and availability of oxidants[84]. 3. Chemical composition of snow and ice, e.g., sea ice contains more chloride that suppresses photoreduction than land ice[85].   **Post deposition**   1. Thawing and percolation of ice and glaciers[86] lead to the loss of the accumulated Hg. 2. Ice and snow density differences due to compression. | 4 |  |  |  | 1  2  3  5 |
| Peat | **Pre-deposition**   1. Anthropogenic/natural disturbances, e.g., peat burning. 2. Peatland types: minerogenic peatland receives Hg from terrestrial water inputs and atmospheric deposition, and ombrotrophic peatland receives Hg only from atmospheric deposition. 3. Morphology of peat: Hummocks and hollows tend to lose and retain Hg[73]. 4. Retention by and bioaccumulation in mire vegetations may lead to elevated flux in the surface layers[87, 88]. 5. Change of mire vegetations affects Hg concentration levels in peat, generally grassland herbs < trees and shrubs < aquatic macrophytes < Sphagnum < mosses < lichens < fungi[89].   **Post-deposition**   1. High (low) peat bulk density and low (high) permeability lead to peaks (humps) of accumulated Hg[90]. 2. Organic matter accumulates Hg[55, 56, 91, 92]. 3. High humidity levels (net surface moisture) provide anaerobic conditions and enhance methylation, eventually leading to the loss of MeHg from peat[58, 59]. 4. Change of hydrological conditions, e.g., extended drought events loss Hg from peatland[93]. 5. High sulfate deposition could change the microbial and chemical environment of peat and eventually reduce the evasion of Hg to the atmosphere[94, 95]. 6. Higher microbial decomposition of peat (thinning of peat), due to concurrent oxygenation of deeper peat layer[96], may release preserved heavy metals. 7. Annual freezing and thawing of peat active layer move Hg downwards[57, 97]. 8. In diagenetic processes, Hg adheres to and moves with oxides and hydroxides (Fe, Mn) with higher redox potential in peat[73]. | 1  2  3  13 | 6 | 7 (some) | 5 | 4  8  9  10  11  12 |
| Lake sediments | **Pre-deposition**   1. Bio-disturbance, e.g., change in lake ecosystem productivity[49, 50, 98], and bird excrement provided Hg inputs to lakes[99]. 2. Melting glaciers and ice provide extra Hg inputs[52, 86]. 3. A larger ratio between catchment areas and lake areas indicates a larger amount of Hg input from the catchment to lake sediments[100]. 4. Natural or manmade disruption in the catchment, e.g., forest fire and wood clearing in catchment areas. 5. Change of catchment land cover affects Hg fluxes in lakes, e.g., dense forests in catchment increase Hg fluxes in lakes[101]. 6. Catchment legacy Hg input[102-104]. 7. Change of Hg input due to change in lake water budget, including surface inflows and outflows and groundwater[87, 105]. 8. Lake surface evasion of Hg to the atmosphere[47, 77, 106]. 9. Elevation affects the supply of atmospheric Hg deposition[65, 107].   **Post-deposition**   1. Sediment bulk density. 2. Sediment focusing heightens Hg fluxes in lake sediments[108]. 3. Sediment texture/grain sizes, e.g., clay and fine sizes accumulate more heavy metals. 4. Organic matter content in sediments. 5. Hg dilutions by natural lithogenic components in sediments or erosions from the catchment[65]. 6. In diagenetic processes, Hg adheres to and moves with oxides and hydroxides (Fe, Mn) with higher redox potential in sediments[107]. 7. Oxygen levels of water. Anoxic and suboxic environments lead to reduction conditions, releasing Fe and Mn, as well as the adsorbed metals to the water (Gawel2014). 8. The acidity of lake water, e.g., low pH of water tends to release Hg to the water. 9. Bio-disturbance at water-sediment interface. 10. Lake water level change disrupts sedimentation hence affecting metal accumulation[105]. | 4  7  15 | 10 | 11 (some)  13 (some) | 3  5  9 | 1  2  5  6  8  12  14  16  17  18  19 |
| Marine sediments | **Pre-deposition**   1. River Hg input[109]. 2. Coastal erosion[42]. 3. Glaciers and permafrost melting[110, 111]. 4. Bio-disturbance, e.g., change in marine ecosystem productivity[45]. 5. Air-water Hg exchange, including evasion[45, 62, 112]. 6. Waterbody stores and buffers Hg deposition[63, 113, 114]. 7. Water current transport of Hg across regions.   **Post-deposition**   1. Sediment bulk density. 2. Sediment focusing heightens Hg fluxes in marine sediments[60]. 3. Sediment texture/ size 4. Sediment is exported to the deep sea with the downward flux of aggregates[61, 62]. 5. Bio-disturbance at water-sediment interface. 6. Diagenetic processes. | 1  13 | 8 |  |  | 2  3  4  5  6  7  9  10  11  12 |

**Table S2** Spatial coverage of natural archive records

| Region | Note | Countries/regions with cores | Country/region count | Core count |
| --- | --- | --- | --- | --- |
| North America |  | Canada, USA | 2 | 104 |
| Europe |  | Czech, Ireland, Norway, Portugal, Spain, Sweden, UK, France | 8 | 24 |
| The Arctic |  | Greenland, Canada | 2 | 24 |
| Latin America | Central America and South America | Peru, Mexico, Argentina, Chile, Ecuador, Jamaica, Barbuda, El Salvador, Falkland Islands | 9 | 20 |
| Central Asia | Elevation areas (>2000 masl) | China, Nepal | 2 | 17 |
| East Asia | China (≤2000 masl) | China, Japan | 2 | 13 |
| Central and southern Africa |  | Lesotho, Tanzania, Uganda | 3 | 7 |
| Oceania |  | New Zealand, Australia | 2 | 5 |
| Southeast Asia |  | Singapore, Philippines | 2 | 3 |
| Antarctica |  | Antarctica | 1 | 3 |
| Indian Ocean |  | Amsterdam | 1 | 1 |
| **Grand Total** |  |  | **34** | **221** |

**Table S3** Descriptive statistics of mercury accumulation flux in natural archives over three centuries. The number of data is the total data points in each type of natural archive during each period without GAM prediction. The Hg flux data for each year within a core is individually counted as a unique data point.

| Type  of  archive | 1700-1799 | | | 1800-1899 | | | 1900-2012 | | | 1980-2012  (Contemporary) | | |
| --- | --- | --- | --- | --- | --- | --- | --- | --- | --- | --- | --- | --- |
|  | Number of data | Mean | SD | Number of data | Mean | SD | Number of data | Mean | SD | Number of data | Mean | SD |
| Lake sediments | 5145 | 0.0090 | 0.0103 | 10194 | 0.0129 | 0.0209 | 16119 | 0.0383 | 0.0891 | 3593 | 0.0548 | 0.1234 |
| Peat | 1094 | 0.0071 | 0.0071 | 1816 | 0.0144 | 0.0172 | 2680 | 0.0302 | 0.0323 | 557 | 0.0331 | 0.0344 |
| Ice | 382 | 0.0002 | 0.0002 | 500 | 0.0004 | 0.0005 | 659 | 0.0026 | 0.0067 | 212 | 0.0015 | 0.0043 |
| Marine sediments | 337 | 0.0145 | 0.0196 | 966 | 0.0290 | 0.0266 | 1924 | 0.0682 | 0.0889 | 290 | 0.1235 | 0.1745 |

**Table S4** Data sources, process methods, and selection of variables for GAM analysis

| Variable type | Factor type | Long name | Used in GAM? | Short name (only for selected ones) | Data description | Data sources | Validation |
| --- | --- | --- | --- | --- | --- | --- | --- |
| Independent variables | Geographic factors | Longitude |  |  | Longitude of coring location | Literature |  |
|  |  | Latitude |  |  | Latitude of coring location | Literature |  |
|  |  | bathymetric depth or Topographic elevation | √ | *Depth/Elevation* | Elevation (masl) at lake surface for lake cores. Elevation at the core top for ice and peat cores. Ocean depth (negative masl) for marine cores | Literature  SRTM 30 m DEM [115].  We prioritized literature disclosed values over the open-source DEM data. |  |
|  |  | Catchment area vs. lake area | √ | *CA/LA* | The ratio between the catchment area and lake area, only for lake cores | Literature  SRTM 30 m DEM [115]. We prioritized literature disclosed values than estimated from open-source DEM data |  |
|  | Environmental factors | Surface albedo |  |  |  | MERRA-2  2°×2.5° | MERRA-2 data were reanalysis products based on satellite observations and these data were evaluated, validated and updated with ground and aircraft observations [116, 117] |
|  |  | Ice-covered fraction of tile |  |  |  |  |  |
|  |  | Leaf area index |  |  |  |  |  |
|  |  | Precipitation | √ | *Precipitation* | Annual mean precipitation (mm) at the respective 2°×2.5° grids of coring locations |  |  |
|  |  | 2-meter specific humidity |  |  |  |  |  |
|  |  | Surface velocity scale |  |  |  |  |  |
|  |  | Temperature | √ | *Temperature* | Annual mean air temperature (°C) at 2 meters above ground at the respective 2°×2.5°grids of coring locations |  |  |
|  |  | Greenness fraction | √ | *Greenness* | Annual mean green colour area percentage at the respective 2°×2.5°grids of coring locations |  |  |
|  | Emission related factors | Local anthropogenic emissions | √ | *Local_AntEmit* | The sum of the annual anthropogenic elemental Hg, anthropogenic divalent Hg, and anthropogenic particulate Hg at the respective 0.1°×0.1° grids of coring locations (kg). | EDGAR  0.1°×0.1° | The uncertainty of EDGAR Hg emission data derives from various parts, including activity data, emission factors, Hg removal efficiency, and gridding [27, 118]. We found that the locations of 13 out of 199 cores in the database were assigned with likely erroneous Hg emissions in EDGAR (See Dataset S1) |
|  |  | Global anthropogenic emissions |  |  |  |  |  |
|  |  | Local re-emissions |  |  |  | GEOS-Chem output  2°×2.5° |  |
|  |  | Local total emissions |  |  |  |  |  |
|  |  | Local non-anthropogenic emissions | √ | *Local_nonAntEmit* | The sum of the annual ocean emission, land re-emission, natural land source, biomass burning, vegetation emissions, soil emissions, and snow emissions at the respective 2°×2.5° grids of coring locations (kg). |  |  |
|  |  | Global non-anthropogenic emissions |  |  |  |  |  |
|  |  | Global re-emissions |  |  |  |  |  |
|  |  | Global total emissions | √ | *Global_TotEmit* | The sum of the annual anthropogenic emissions and non-anthropogenic emissions at all grids (ton), except the grids of coring locations. |  |  |
| Response variables | Natural archive Hg flux | Hg accumulation fluxes in lake sediments | √ | *Hg-LakeSed* | All natural-archive data from lake cores except those cores found to have possible erroneous local anthropogenic emissions in EDGAR | The database (this study) |  |
|  |  | Hg accumulation fluxes in marine sediments | √ | *Hg-MarineSed* | All natural-archive data from marine cores |  |  |
|  |  | Hg accumulation fluxes in peatlands | √ | *Hg-Peat* | All natural-archive data from peat cores except those found to have possible erroneous local anthropogenic emissions in EDGAR |  |  |
|  |  | Hg accumulation fluxes in ice cores | √ | *Hg-Ice* | All natural-archive data from ice cores, no snow cores included |  |  |
|  |  | Hg accumulation fluxes in all four types of natural archives in the database | √ | *Hg-CoreFlux* | All natural-archive data in the database without excluding any cores. |  |  |

Table S5 GAM 10-fold cross-validation analysis. GAMscale refers to the scale parameter of the GAM model; CV-mse-GAM refers to the Mean Squared Error obtained using cross-validation for GAM analysis.

| GAM analysis | GAMscale | CV-mse-GAM |
| --- | --- | --- |
| Lake cores | 0.0002 | 0.0002 |
| Peat cores | 0.0001 | 0.0001 |
| Marine cores | 0.0000 | 0.0000 |
| Ice cores | 0.0008 | 0.0012 |


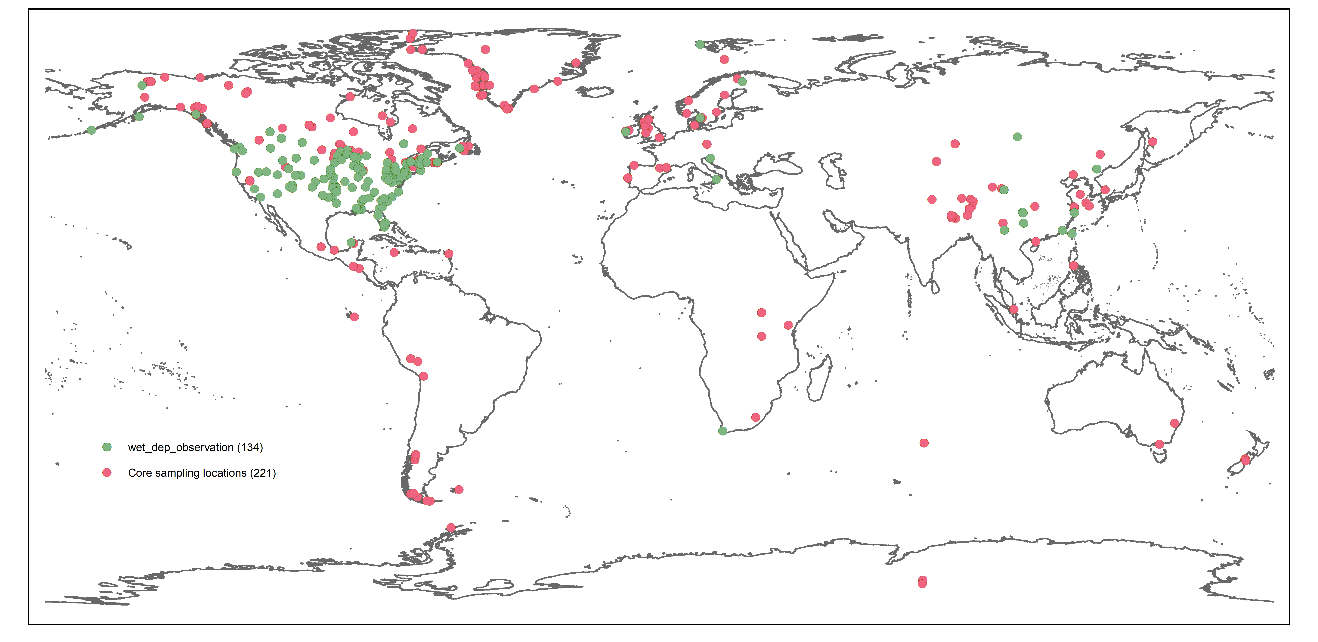


Fig. S1 Spatial distribution of sampling locations of natural archive cores used in this study (SI Dataset S1) and ground monitoring stations of mercury wet deposition (SI Dataset S2).


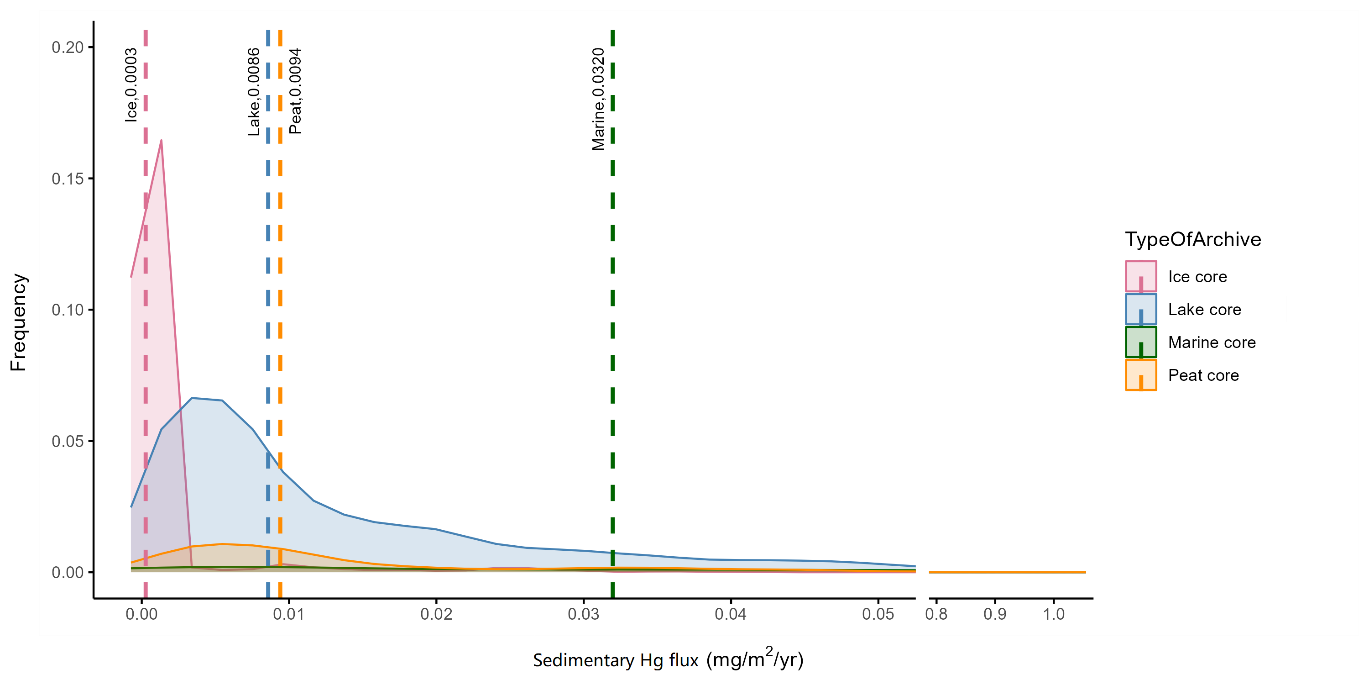
Fig. S2 Data frequency of mercury accumulation fluxes in the four types of natural archive 1700-2012. The dotted lines were median fluxes.


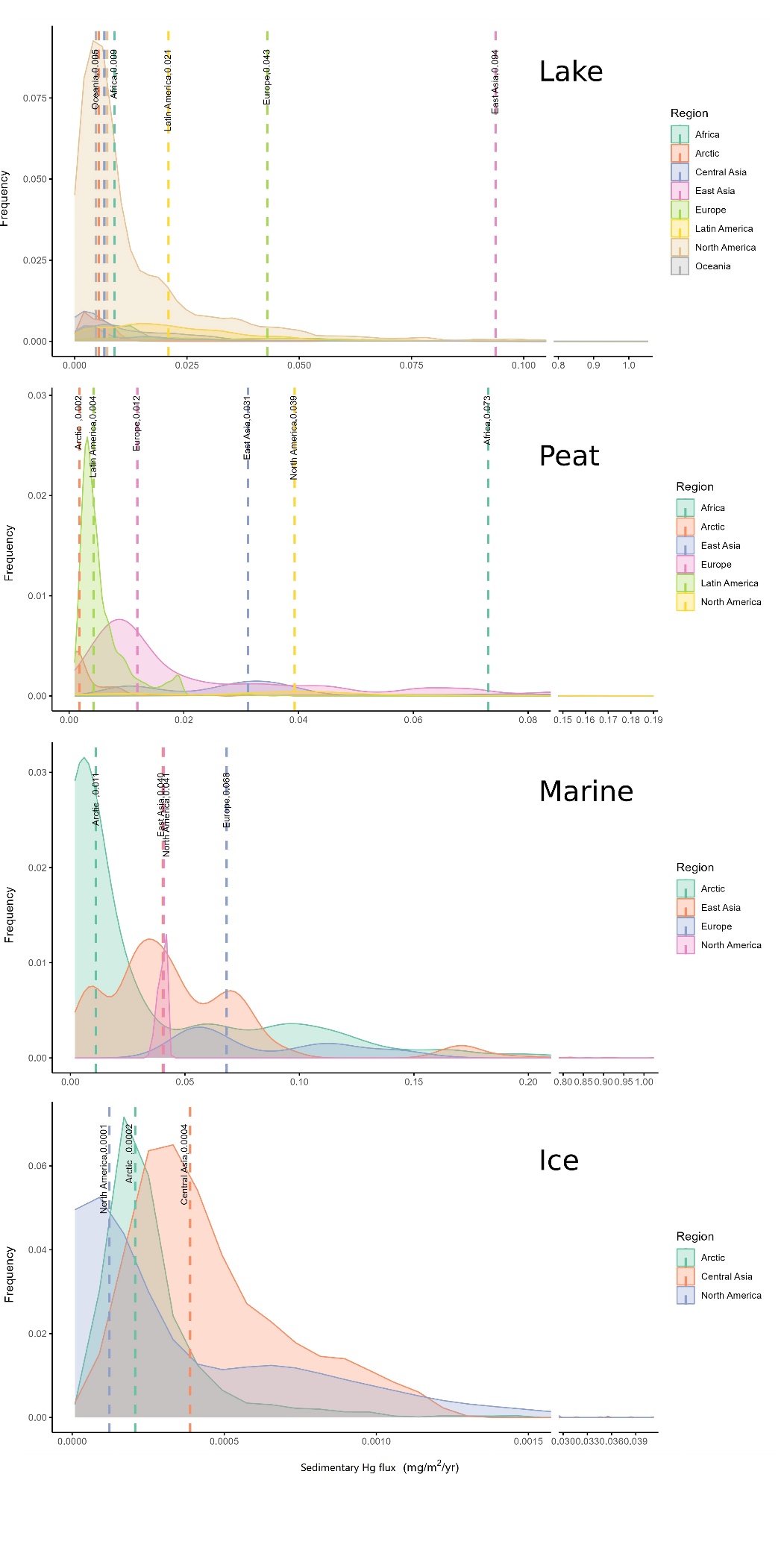
Fig. S3 Data frequency of mercury accumulation fluxes in the four types of natural archive by region 1700-2012. The dotted lines were median fluxes of regions.


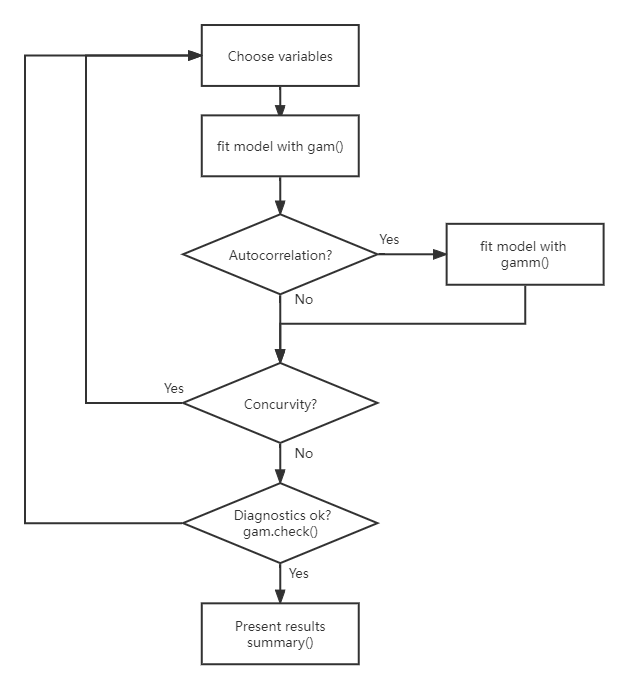


**Fig. S4** GAM analysis flow chart


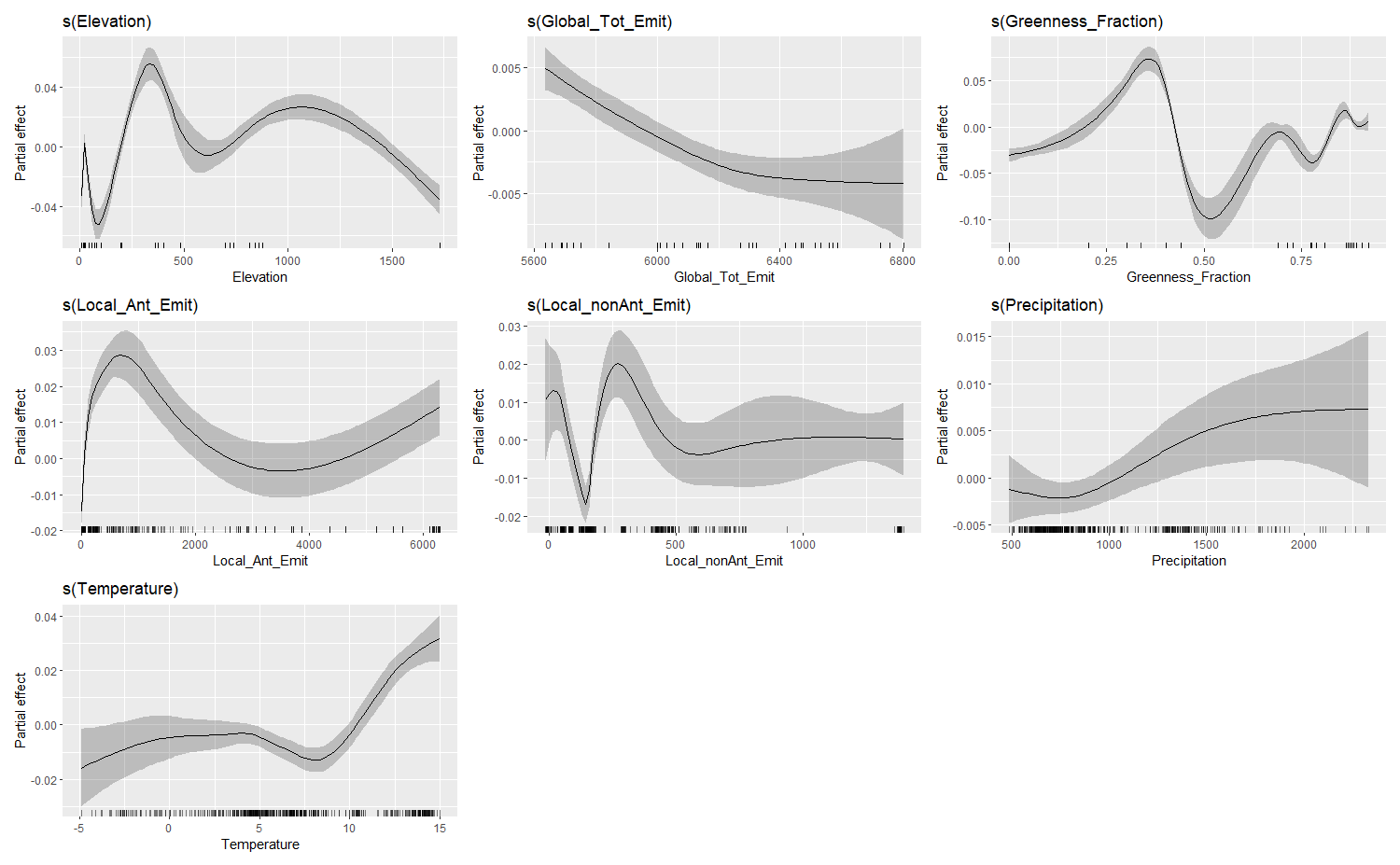


**Fig. S5** Partial effects of variables on the natural-archive Hg fluxes from peat cores.


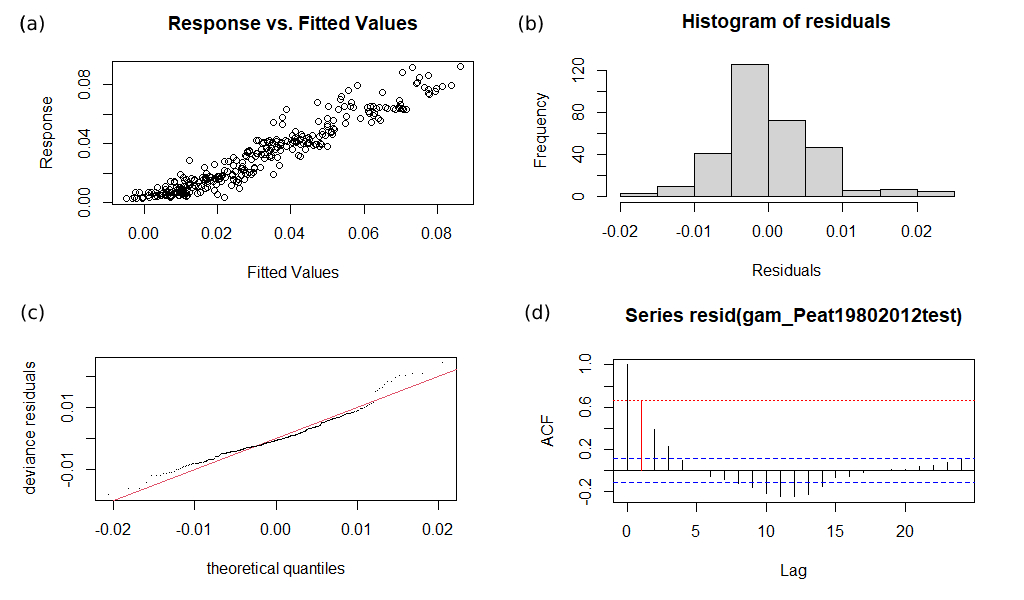


**Fig. S6** GAM result check plots for peat cores. (a) fitted value and natural-archive Hg flux values for peat cores. (b) Histogram of residuals of the fitted values for peat cores. (c) QQ plot for GAM fitted results for peat cores. (d) Autocorrelation of simulated residuals for peat cores.


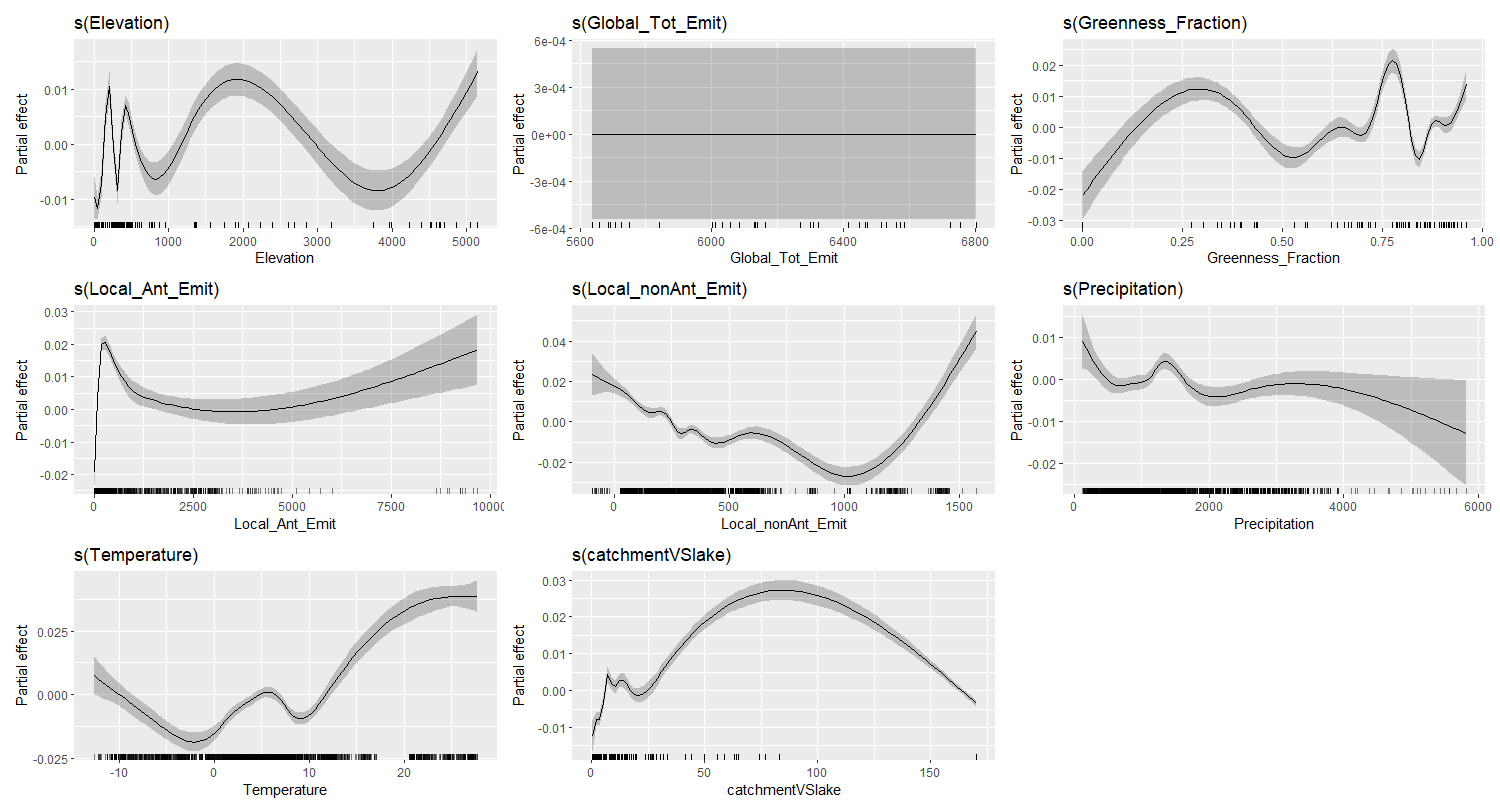


**Fig. S7** Partial effects of variables on the natural-archive Hg fluxes from lake cores.


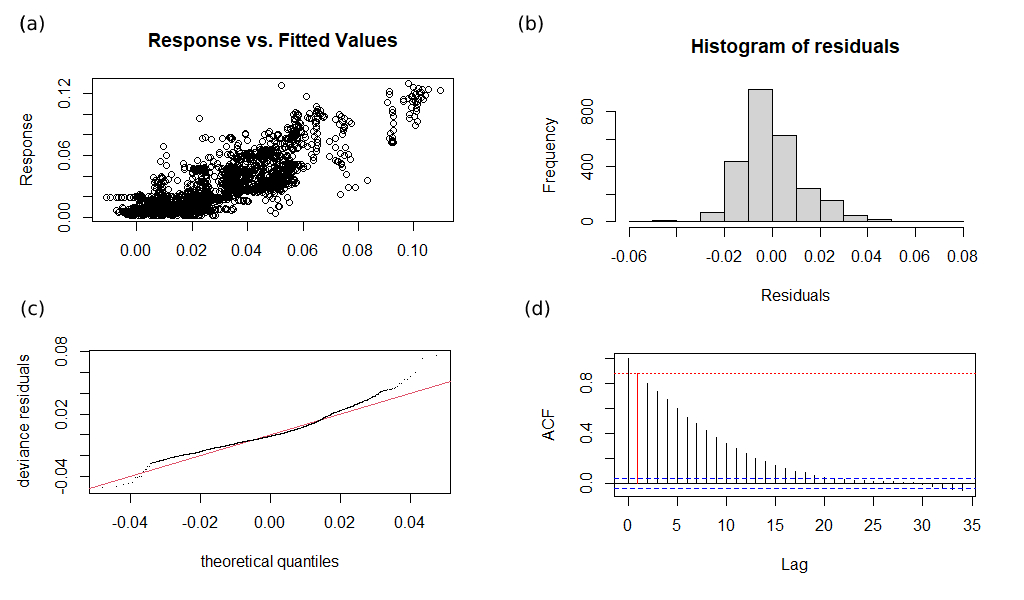


**Fig. S8** GAM result check plots for lake cores. (a) fitted value and natural-archive Hg flux values for lake cores. (b) Histogram of residuals of the fitted values for lake cores. (c) QQ plot for GAM fitted results for lake cores. (d) Autocorrelation of simulated residuals for lake cores.


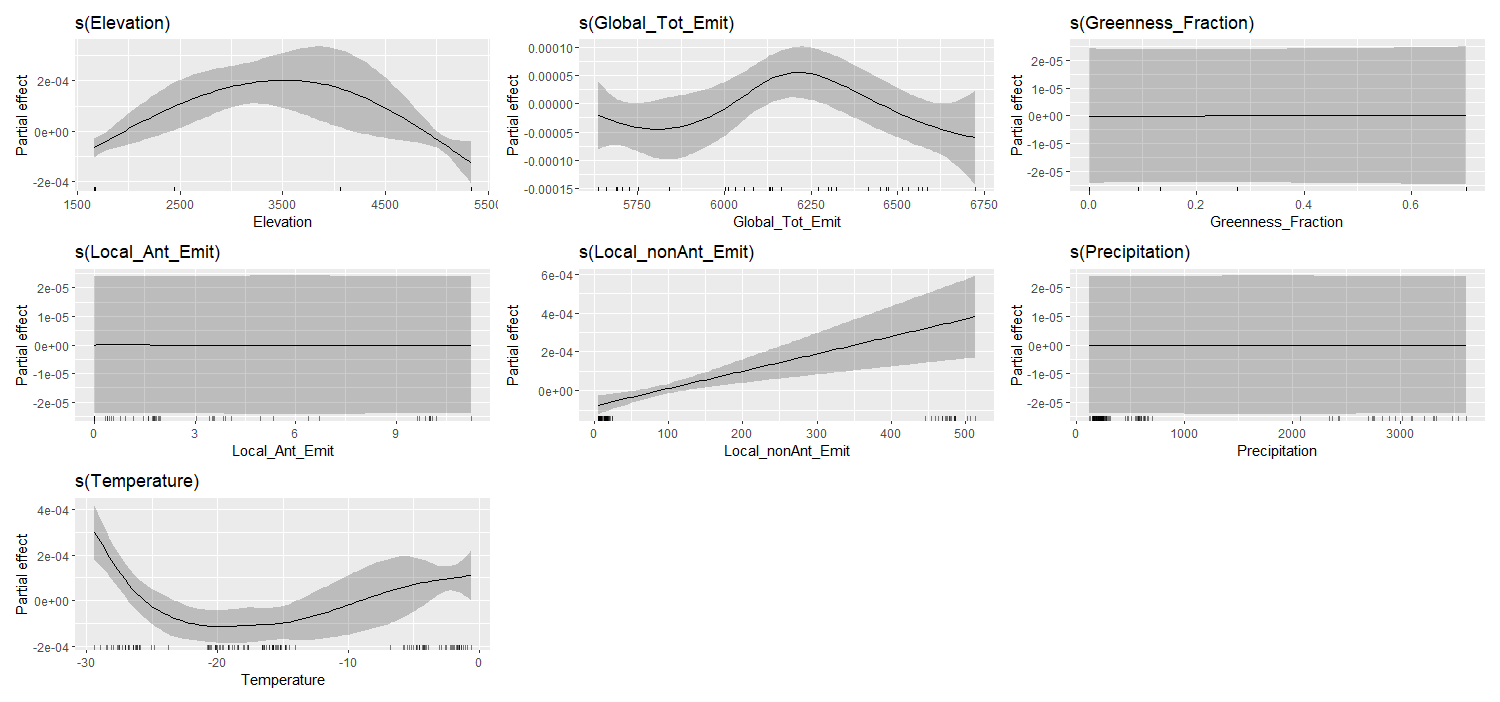


**Fig. S9** Partial effects of variables on the natural-archive Hg fluxes from ice cores (excluding snow cores (2 cores)).


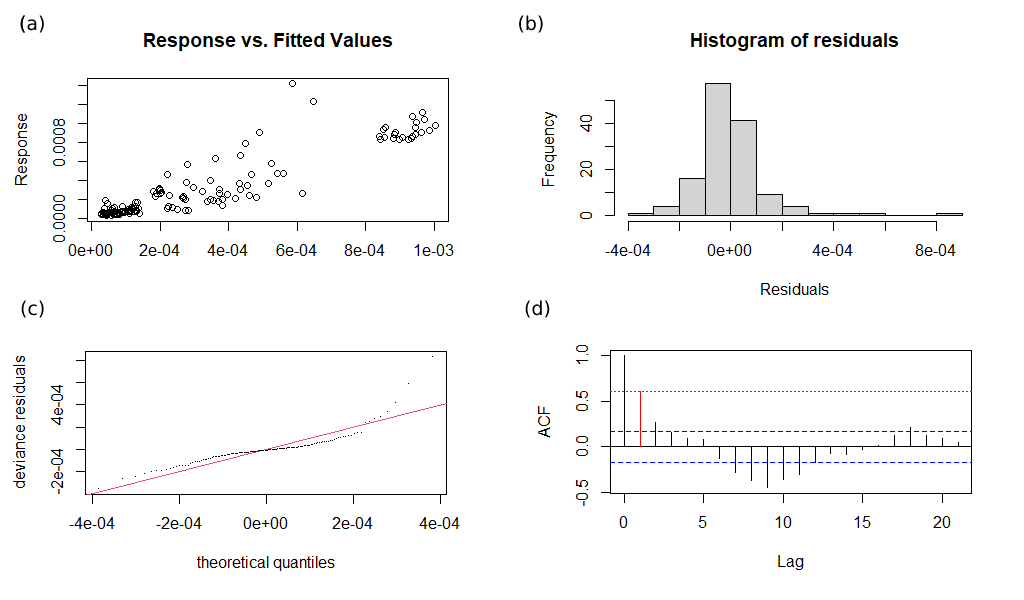


**Fig. S10** GAM result check plots for ice cores. (a) fitted value and natural-archive Hg flux values for ice cores. The scattered samples in the central plot are the Greenland ice core [66]. If removing these samples, the GAM model would not converge due to a limited number of ice core samples. Therefore, we keep these samples. (b) Histogram of residuals of the fitted values for ice cores. (c) QQ plot for GAM fitted results for ice cores. (d) Autocorrelation of simulated residuals for ice cores.


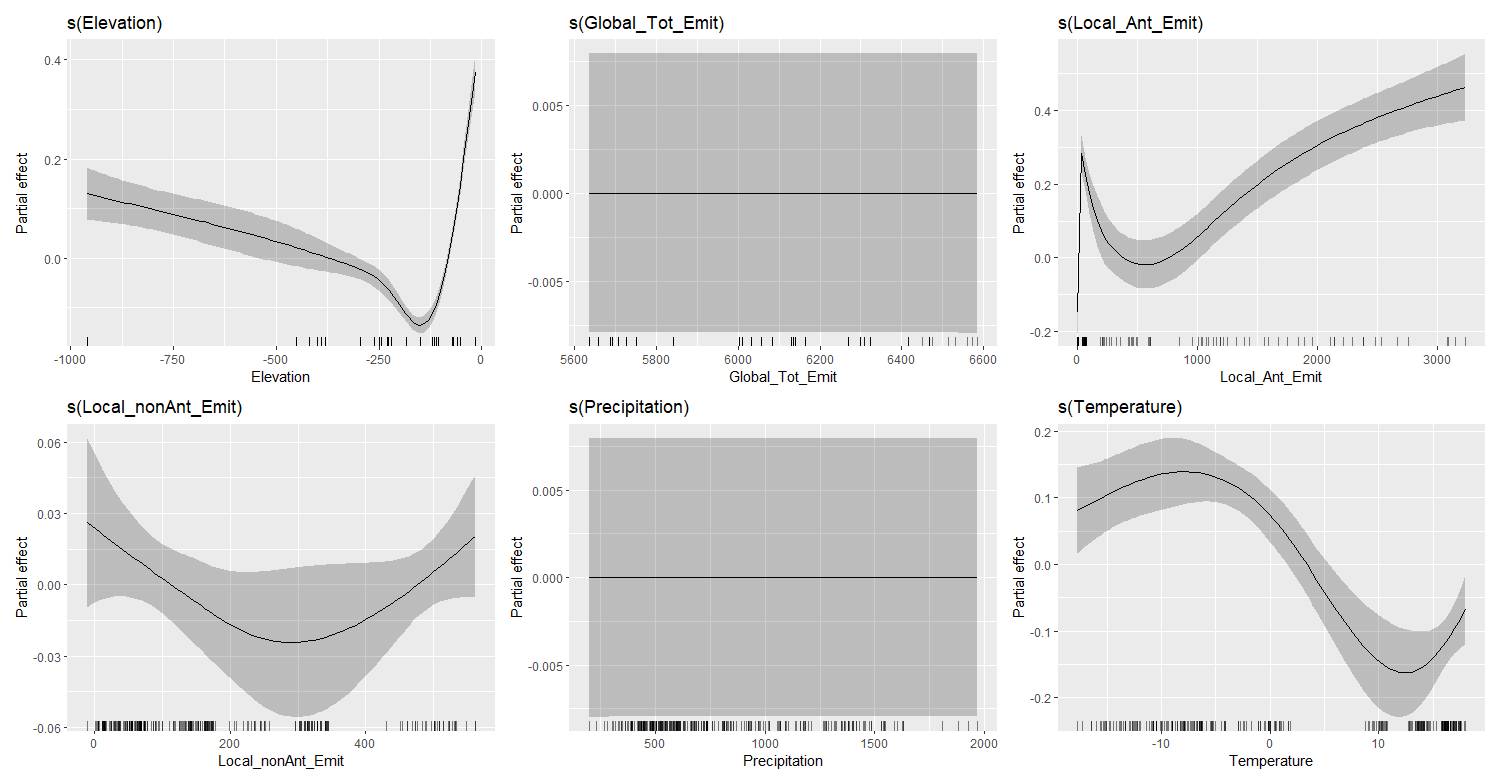


**Fig. S11** Partial effects of variables on the natural-archive Hg fluxes from marine cores.


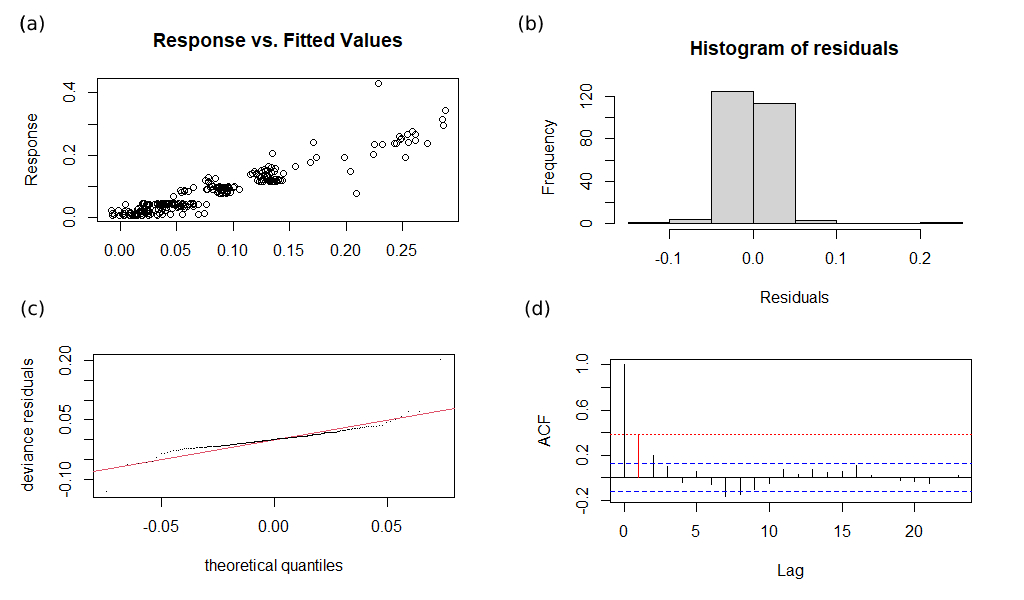


**Fig. S12** GAM result check plots for marine cores. (a) fitted value and natural-archive Hg flux values for marine cores. (b) Histogram of residuals of the fitted values for marine cores. (c) QQ plot for GAM fitted results for marine cores. (d) Autocorrelation of simulated residuals for marine cores.


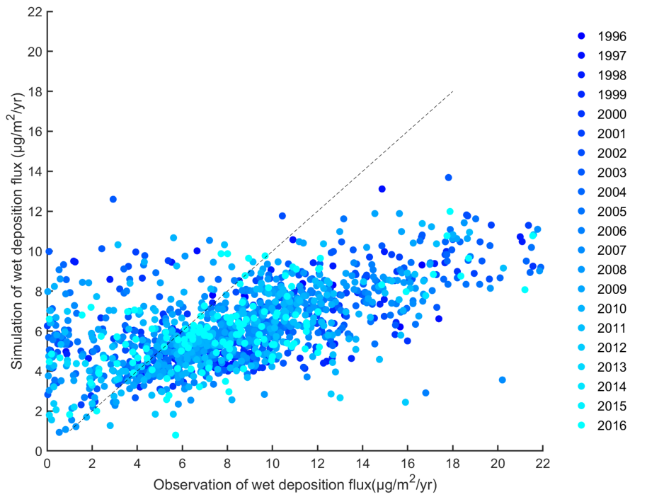


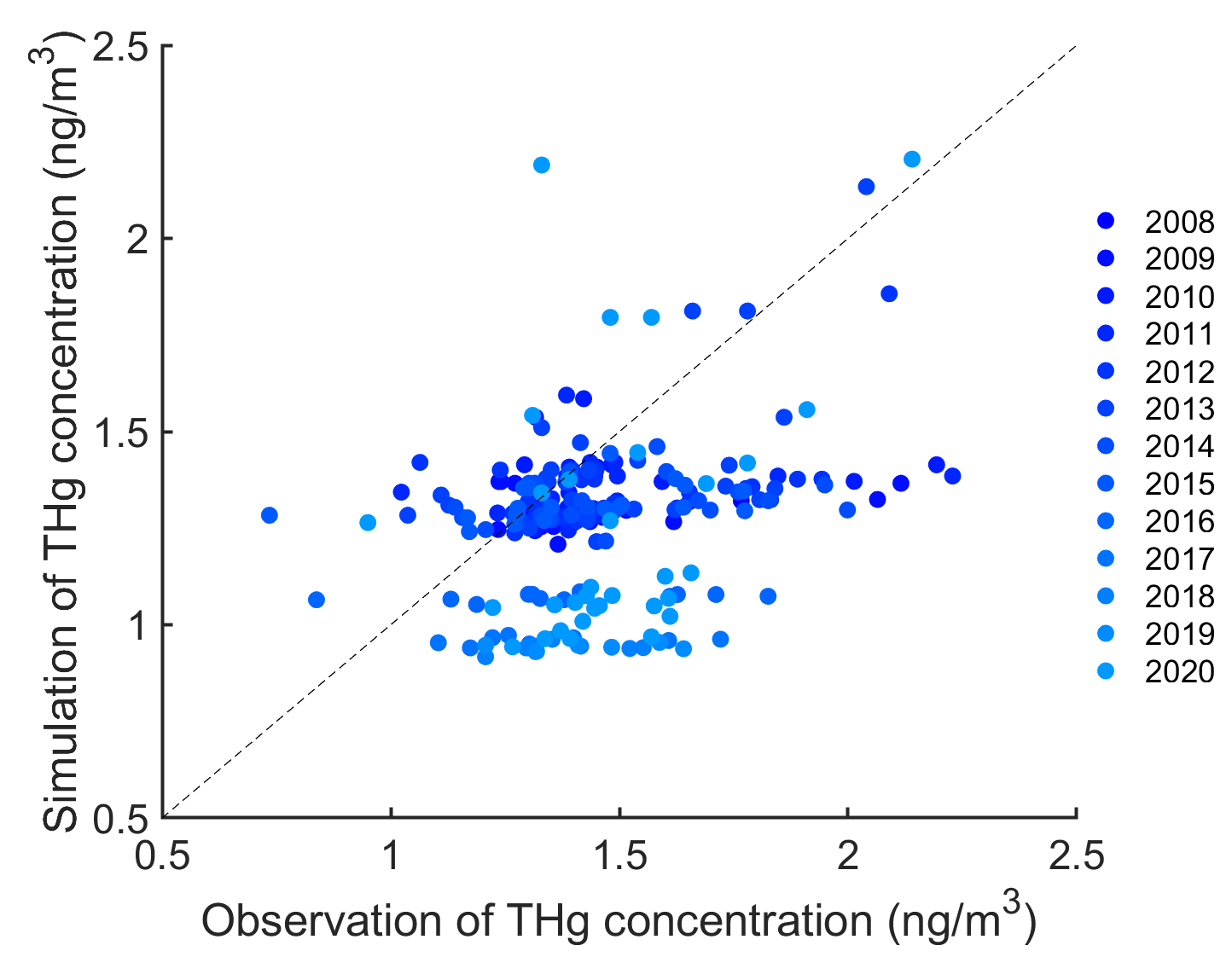


**Fig. S13** GEOS-chem modelled wet depositions (upper) and concentrations (lower) vs ground observation of Hg. For wet depositions, the validation shows a deviation of normalized mean bias ($\frac{\sum_{i} (Simulation_{i}-{Observation}_{i})}{\sum_{i} {Observation}_{i}}$) of -40.2% and normalized mean error ($\frac{\sum_{i} |Simulation_{i}-{Observation}_{i}|}{\sum_{i} {Observation}_{i}}$) of 51.4%. The result shows a general overestimation (underestimation) of the modelled wet deposition below (above) ca. 4 µg/m^2^/yr. For Hg concentrations, the validation shows a normalized mean bias of -12.4% and a normalized mean error of 16.7%, This validation result shows high consistency between the modelled concentrations with the observed which indicates the robustness of the model.


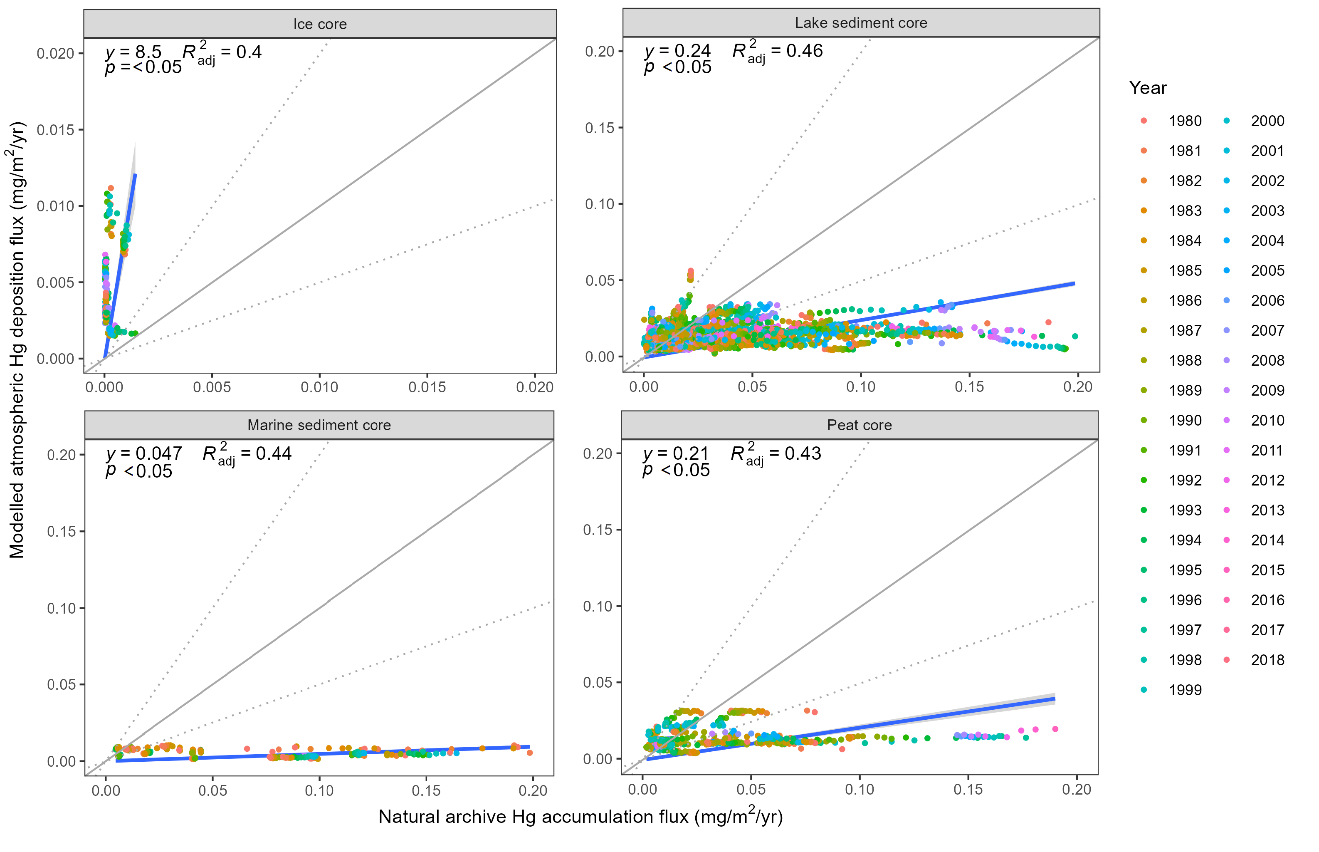


**Fig. S14** Natural archive accumulation Hg fluxes vs GEOS-chem modelled fluxes using data from 1980 to 2020. The solid grey line is a reference line of y=x, dotted grey lines are y = 2x and y = 1/2x, respectively. Blue lines are fitted regression lines using a no-intercept ordinary linear regression model.

**
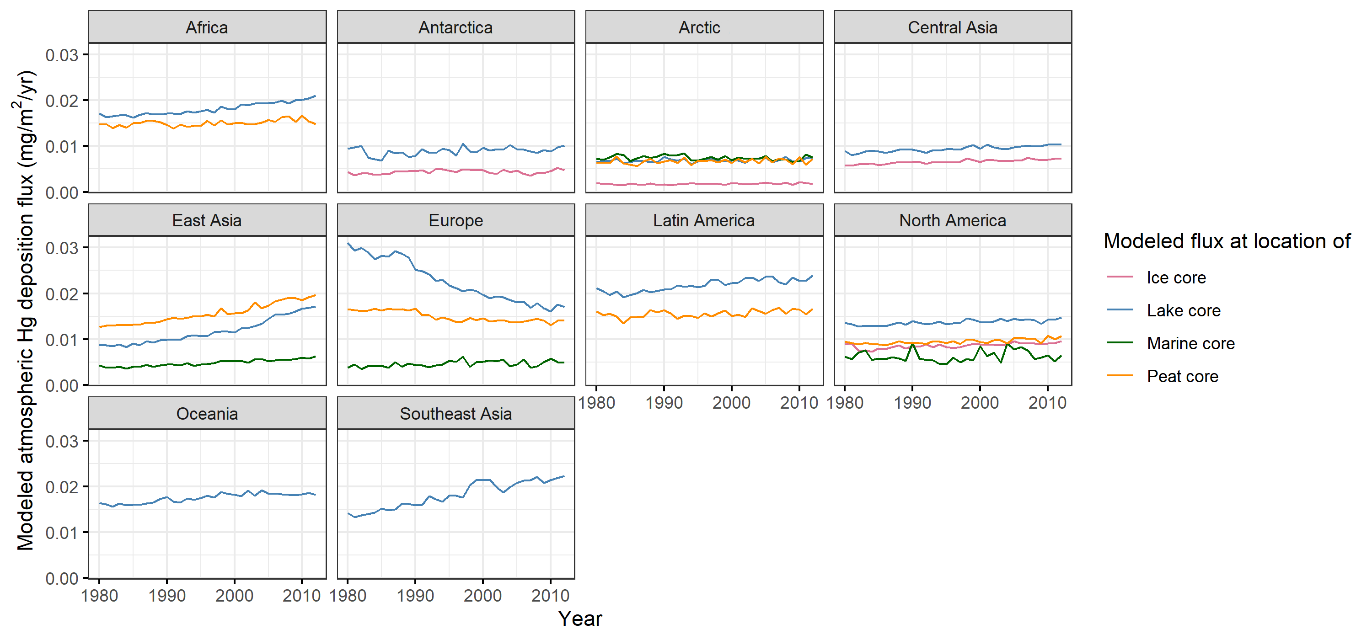
Fig. S15** GEOS-Chem modelled atmospheric Hg deposition at locations of cores by region 1980-2012. Africa refers to Central and Southern Africa, Oceania refers to Australia and New Zealand, Latin America refers to Mexico and the western Andes, and the Arctic refers to Greenland and nearby islands.


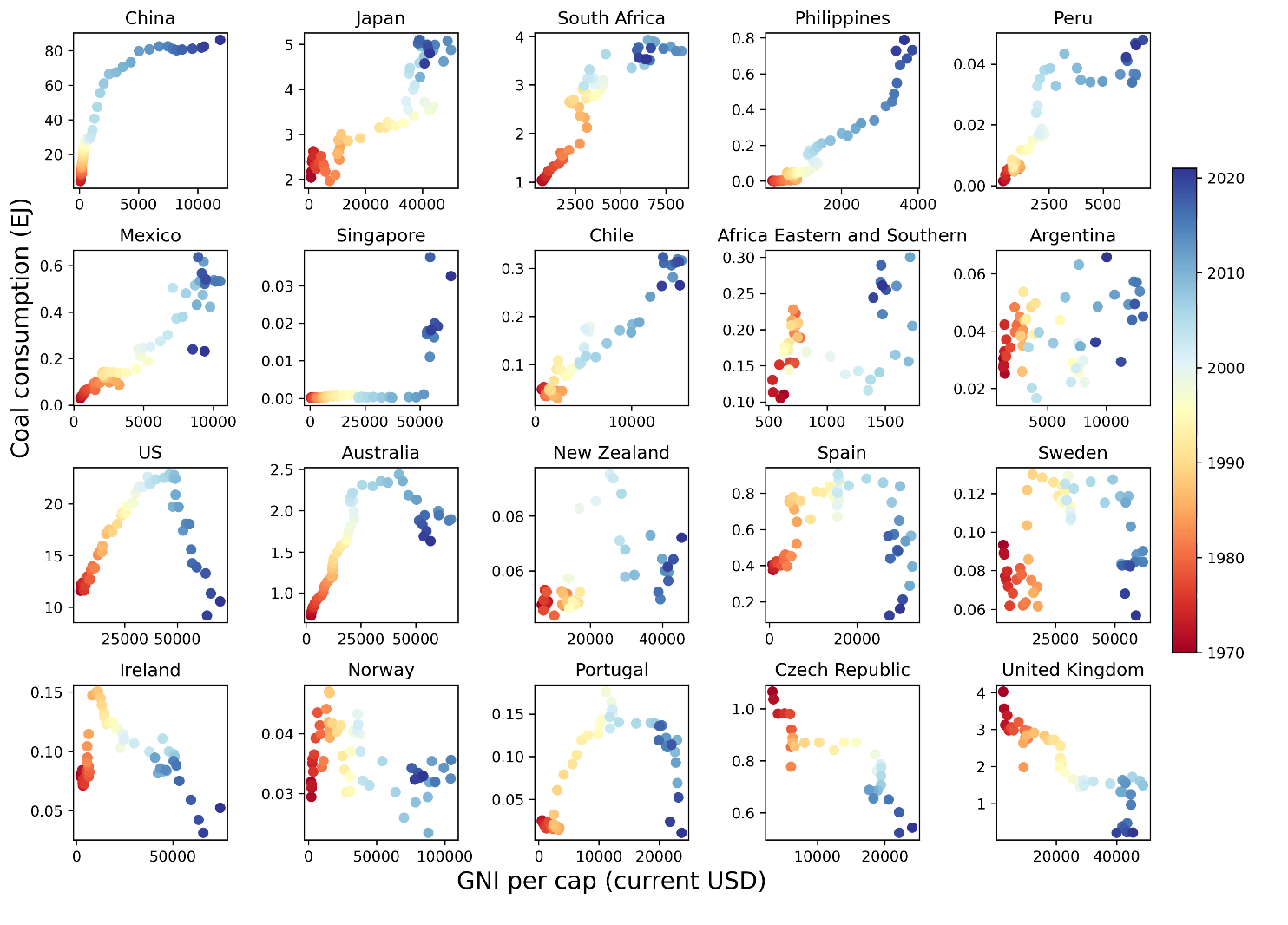


**Fig. S16** Scatter plots of coal consumption (EJ) [119] and economic development indicated by GNI per cap (current USD)[120] between 1970 and 2021 in 20 key countries in the database. The simple plots indicate that East Asian countries including China and Japan, African countries including South Africa, and Latin American countries Peru, Mexico, and Chile, have yet to decouple their coal use from economic development. By contrast, North American countries including the US, and European countries including Spain, Sweden, Ireland, Norway, Portugal, Czech, and the United Kingdom, have already decoupled their coal consumption with economic development.


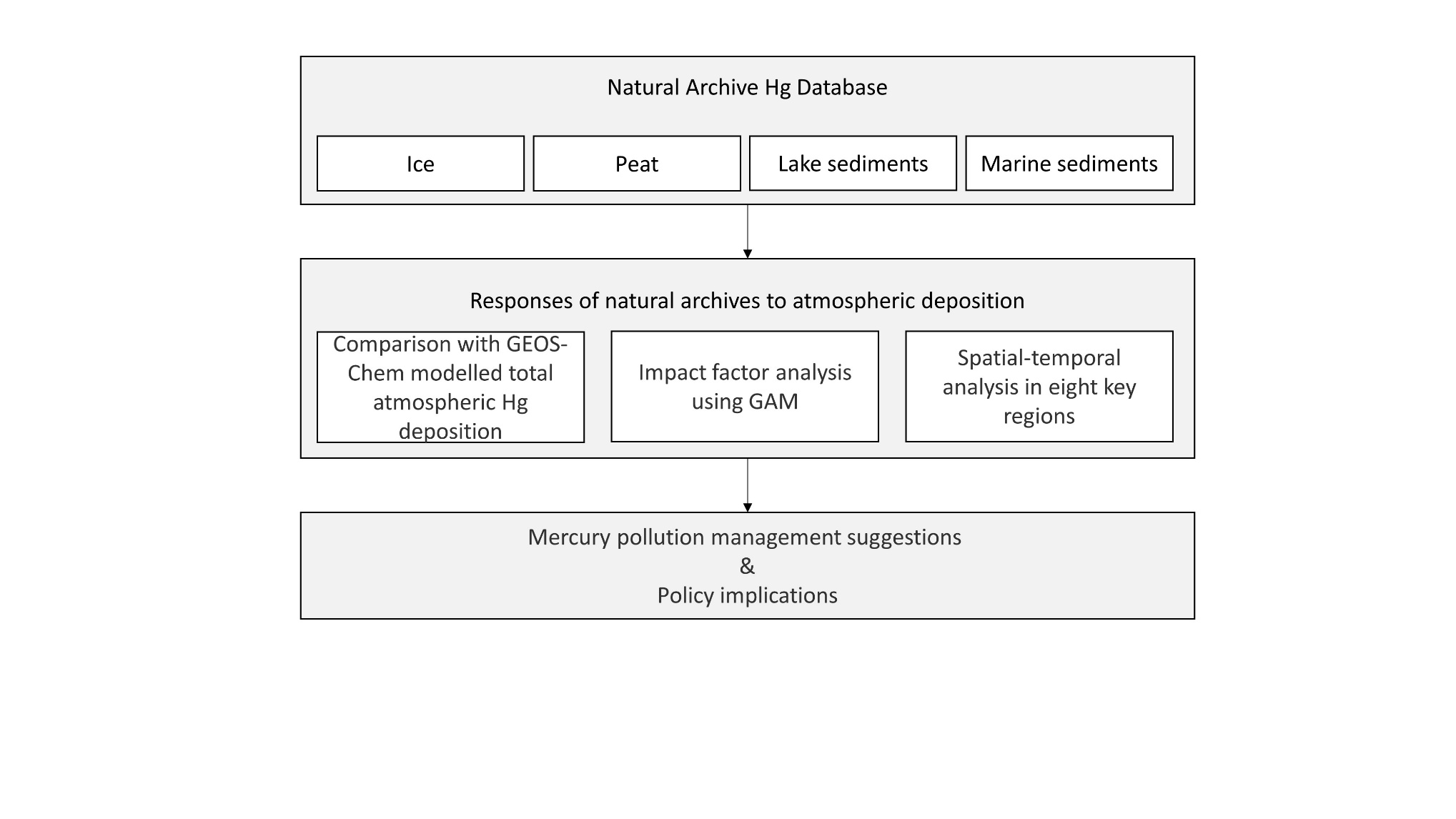


Fig. S 17 The workflow of this study comprises three key steps. Step 1 involves the preparation of natural archive Hg records sourced from ice, peat, lake sediments, and marine sediments. Step 2 focuses on analysing the response of natural archive Hg fluxes to atmospheric Hg depositions. This step is divided into three parts: first, comparing natural archive Hg fluxes with GEOS-Chem modelling results to identify disparities and similarities; second, examining eight key factors that influence changes in natural archive mercury fluxes; and third, conducting spatial-temporal analysis to understand the responses of natural archives in different regions. Step 3 aims to identify key ecosystems and regions for future Hg pollution management and discuss policy implications.

**Dataset S1** **(separate file, SI Natural Archive Database.xlsx)**. Nature-archive mercury accumulation flux database 1700-2012, containing core information, references, and respective Hg accumulation flux data.

**Dataset S2 (separate file, SI Monitoring data.xlsx)**. Ground monitoring of wet deposition and ambient concentration of mercury, containing location information and deposition flux data.

**Dataset S3 (separate file, SI Figure Source Data.xlsx)**. The source data used to plot Figures 1-3 in the manuscript.

# SI References

1. Cooke CA, Martínez-Cortizas A, Bindler R *et al.* Environmental archives of atmospheric Hg deposition – A review. *Sci Total Environ*. 2020; **709**: 134800. doi: 10.1016/j.scitotenv.2019.134800

2. Li F, Ma C, Zhang P. Mercury Deposition, Climate Change and Anthropogenic Activities: A Review. *Frontiers in Earth Science*. 2020; **8**. doi: 10.3389/feart.2020.00316

3. Li C, Sonke JE, Le Roux G *et al.* Unequal Anthropogenic Enrichment of Mercury in Earth’s Northern and Southern Hemispheres. *ACS Earth and Space Chemistry*. 2020; **4**(11): 2073-2081. doi: 10.1021/acsearthspacechem.0c00220

4. Engstrom DR, Fitzgerald WF, Cooke CA *et al.* Atmospheric Hg Emissions from Preindustrial Gold and Silver Extraction in the Americas: A Reevaluation from Lake-Sediment Archives. *Environ Sci Technol*. 2014; **48**(12): 6533-6543. doi: 10.1021/es405558e

5. Goodsite ME, Outridge PM, Christensen JH *et al.* How well do environmental archives of atmospheric mercury deposition in the Arctic reproduce rates and trends depicted by atmospheric models and measurements? *Sci Total Environ*. 2013; **452-453**: 196-207. doi: 10.1016/j.scitotenv.2013.02.052

6. Binford MW. Calculation and uncertainty analysis of 210Pb dates for PIRLA project lake sediment cores. *J Paleolimnol*. 1990; **3**(3): 253-267.

7. Stuiver M, Reimer PJ, Bard E *et al.* INTCAL98 radiocarbon age calibration, 24,000–0 cal BP. *Radiocarbon*. 1998; **40**(3): 1041-1083.

8. Gelaro R, McCarty W, Suárez MJ *et al.* The modern-era retrospective analysis for research and applications, version 2 (MERRA-2). *J Clim*. 2017; **30**(14): 5419-5454.

9. Selin NE, Jacob DJ, Yantosca RM *et al.* Global 3‐D land‐ocean‐atmosphere model for mercury: Present‐day versus preindustrial cycles and anthropogenic enrichment factors for deposition. *Global biogeochemical cycles*. 2008; **22**(2): 1-13.

10. Schroeder WH, Munthe J. Atmospheric mercury—An overview. *Atmospheric Environment*. 1998; **32**(5): 809-822. doi: <https://doi.org/10.1016/S1352-2310(97)00293-8>

11. Selin NE, Jacob DJ, Park RJ *et al.* Chemical cycling and deposition of atmospheric mercury: Global constraints from observations. *Journal of Geophysical Research Atmospheres*. 2007; **112**.

12. Sprovieri F, Pirrone N, Bencardino M *et al.* Five-year records of mercury wet deposition flux at GMOS sites in the Northern and Southern hemispheres. *Atmos Chem Phys*. 2017; **17**(4): 2689-2708. doi: 10.5194/acp-17-2689-2017

13. Wesely ML. Parameterization of surface resistances to gaseous dry deposition in regional-scale numerical models. *Atmospheric Environment*. 1989; **23**(supp-S): 1293-1304.

14. Selin NE. Global 3-D land-ocean-atmosphere model for mercury: Present-day versus preindustrial cycles and anthropogenic enrichment factors for deposition. *Global Biogeochemical Cycles*. 2008; **22**.

15. Wesely ML, Hicks BB. A review of the current status of knowledge on dry deposition. *Atmospheric Environment*. 2000; **34**(12): 2261-2282. doi: <https://doi.org/10.1016/S1352-2310(99)00467-7>

16. Zhang L, Brook JR, Vet R. A revised parameterization for gaseous dry deposition in air-quality models. *Atmos Chem Phys*. 2003; **3**(6): 2067-2082. doi: 10.5194/acp-3-2067-2003

17. Zhang L, Zhang GC, Zhou PS *et al.* A Review of Dry Deposition Schemes for Speciated Atmospheric Mercury. *Bull Environ Contam Toxicol*. 2023; **110**(1): 16. doi: ARTN 16

10.1007/s00128-022-03641-0

18. Zhou J, Bollen SW, Roy EM *et al.* Comparing ecosystem gaseous elemental mercury fluxes over a deciduous and coniferous forest. *Nature Communications*. 2023; **14**(1): 2722. doi: ARTN 2722

10.1038/s41467-023-38225-x

19. Duan L, Sun XH, Luo Y *et al.* Soil-atmosphere exchange of gaseous elemental mercury in three subtropical forests with different substrate Hg concentrations. *Atmos Environ*. 2021; **244**: 117869. doi: ARTN 117860

10.1016/j.atmosenv.2020.117860

20. Zhang L, Zhou PS, Cao SZ *et al.* Atmospheric mercury deposition over the land surfaces and the associated uncertainties in observations and simulations: a critical review. *Atmospheric Chemistry and Physics*. 2019; **19**(24): 15587-15608. doi: 10.5194/acp-19-15587-2019

21. Sommar J, Osterwalder S, Zhu W. Recent advances in understanding and measurement of Hg in the environment: Surface-atmosphere exchange of gaseous elemental mercury (Hg). *Sci Total Environ*. 2020; **721**. doi: ARTN 137648

10.1016/j.scitotenv.2020.137648

22. Zhu W, Lin CJ, Wang X *et al.* Global observations and modeling of atmosphere-surface exchange of elemental mercury: a critical review. *Atmospheric Chemistry and Physics*. 2016; **16**(7): 4451-4480. doi: 10.5194/acp-16-4451-2016

23. Yu K, Keller CA, Jacob DJ *et al.* Errors and improvements in the use of archived meteorological data for chemical transport modeling: an analysis using GEOS-Chem v11-01 driven by GEOS-5 meteorology. *Geosci Model Dev*. 2018; **11**(1): 305-319. doi: 10.5194/gmd-11-305-2018

24. Horowitz HM, Jacob DJ, Zhang Y *et al.* A new mechanism for atmospheric mercury redox chemistry: implications for the global mercury budget. *Atmospheric Chemistry and Physics*. 2017; **17**(10): 6353-6371. doi: 10.5194/acp-17-6353-2017

25. Liu K, Wu Q, Wang L *et al.* Measure-Specific Effectiveness of Air Pollution Control on China’s Atmospheric Mercury Concentration and Deposition during 2013–2017. *Environ Sci Technol*. 2019; **53**(15): 8938-8946. doi: 10.1021/acs.est.9b02428

26. Larsen K. *GAM: The Predictive Modeling Silver Bullet*. 2015.

27. Muntean M, Janssens-Maenhout G, Song S *et al.* Evaluating EDGARv4.tox2 speciated mercury emissions ex-post scenarios and their impacts on modelled global and regional wet deposition patterns. *Atmos Environ*. 2018; **184**: 56-68. doi: 10.1016/j.atmosenv.2018.04.017

28. Menard S. *Applied logistic regression analysis*: Sage, 2002.

29. Gareth J, Daniela W, Trevor H *et al.* *An introduction to statistical learning: with applications in R*: Springer, 2013.

30. McCullagh P, Nelder J. *Generalized linear models*: Chapman and Hill, 1989.

31. Hastie T, Tibshirani R, Friedman JH *et al.* *The elements of statistical learning: data mining, inference, and prediction*: Springer, 2009.

32. DataCamp. *CVgam: Cross-validation estimate of accuracy from GAM model fit*2020.

33. Pedersen EJ, Miller DL, Simpson GL *et al.* Hierarchical generalized additive models in ecology: an introduction with mgcv. *PeerJ*. 2019; **7**: e6876.

34. Gilbert RO. *Statistical methods for environmental pollution monitoring*: John Wiley & Sons, 1987.

35. Streets DG, Horowitz HM, Jacob DJ *et al.* Total Mercury Released to the Environment by Human Activities. *Environ Sci Technol*. 2017; **51**(11): 5969-5977. doi: 10.1021/acs.est.7b00451

36. UNEP. *Global Mercury Assessment 2018*. Geneva, Switzerland: United Nations Environment Programme; 2019.

37. Matsumura K, Kawase K, Takeya K. Observation of sublimation of ice using terahertz spectroscopy. *Royal Society Open Science*. 2020; **7**(9): 192083. doi: doi:10.1098/rsos.192083

38. Huang J, Kang S, Zhang Q *et al.* Spatial distribution and magnification processes of mercury in snow from high-elevation glaciers in the Tibetan Plateau. *Atmos Environ*. 2012; **46**: 140-146. doi: 10.1016/j.atmosenv.2011.10.008

39. DiMento BP, Mason RP, Brooks S *et al.* The impact of sea ice on the air-sea exchange of mercury in the Arctic Ocean. *Deep Sea Research Part I: Oceanographic Research Papers*. 2019; **144**: 28-38. doi: 10.1016/j.dsr.2018.12.001

40. Overeem I, Hudson BD, Syvitski JPM *et al.* Substantial export of suspended sediment to the global oceans from glacial erosion in Greenland. *Nature Geoscience*. 2017; **10**(11): 859-863. doi: 10.1038/ngeo3046

41. Fox-Kemper B, H.T. Hewitt, C. Xiao, G. Aðalgeirsdóttir, S.S. Drijfhout, T.L. Edwards, N.R. Golledge, M. Hemer, R.E. Kopp, G. Krinner, A. Mix, D. Notz, S. Nowicki, I.S. Nurhati, L. Ruiz, J.-B. Sallée, A.B.A. Slangen, and Y. Yu. *Ocean, Cryosphere and Sea Level Change*. Cambridge, United Kingdom and New York, NY, USA2021.

42. Meng M, Sun R-y, Liu H-w *et al.* An Integrated Model for Input and Migration of Mercury in Chinese Coastal Sediments. *Environ Sci Technol*. 2019; **53**(5): 2460-2471. doi: 10.1021/acs.est.8b06329

43. Loranty MM, Lieberman-Cribbin W, Berner LT *et al.* Spatial variation in vegetation productivity trends, fire disturbance, and soil carbon across arctic-boreal permafrost ecosystems. *ENVIRONMENTAL RESEARCH LETTERS*. 2016; **11**(9). doi: 10.1088/1748-9326/11/9/095008

44. Trombetta T, Vidussi F, Mas S *et al.* Water temperature drives phytoplankton blooms in coastal waters. *PLOS ONE*. 2019; **14**(4). doi: 10.1371/journal.pone.0214933

45. Zhang Y, Zhang P, Song Z *et al.* An updated global mercury budget from a coupled atmosphere-land-ocean model: 40% more re-emissions buffer the effect of primary emission reductions. *One Earth*. 2023; **6**(3): 316-325. doi: 10.1016/j.oneear.2023.02.004

46. Zhou J, Obrist D, Dastoor A *et al.* Vegetation uptake of mercury and impacts on global cycling. *Nature Reviews Earth & Environment*. 2021; **2**(4): 269-284. doi: 10.1038/s43017-021-00146-y

47. Southworth G, Lindberg S, Hintelmann H *et al.* Evasion of added isotopic mercury from a northern temperate lake. *Environmental Toxicology and Chemistry: An International Journal*. 2007; **26**(1): 53-60. doi: 10.1897/06-148R.1

48. Yang H, Engstrom DR, Rose NL. Recent Changes in Atmospheric Mercury Deposition Recorded in the Sediments of Remote Equatorial Lakes in the Rwenzori Mountains, Uganda. *Environ Sci Technol*. 2010; **44**(17): 6570-6575. doi: 10.1021/es101508p

49. Creed IF, Bergstrom AK, Trick CG *et al.* Global change-driven effects on dissolved organic matter composition: Implications for food webs of northern lakes. *Global Change Biol*. 2018; **24**(8): 3692-3714. doi: 10.1111/gcb.14129

50. Rydberg J, Klaminder J, Rosén P *et al.* Climate driven release of carbon and mercury from permafrost mires increases mercury loading to sub-arctic lakes. *Sci Total Environ*. 2010; **408**(20): 4778-4783. doi: <https://doi.org/10.1016/j.scitotenv.2010.06.056>

51. Cooke CA, Wolfe AP, Michelutti N *et al.* A Holocene Perspective on Algal Mercury Scavenging to Sediments of an Arctic Lake. *Environ Sci Technol*. 2012; **46**(13): 7135-7141. doi: 10.1021/es3003124

52. Zhu T, Wang X, Lin H *et al.* Accumulation of Pollutants in Proglacial Lake Sediments: Impacts of Glacial Meltwater and Anthropogenic Activities. *Environ Sci Technol*. 2020; **54**(13): 7901-7910. doi: 10.1021/acs.est.0c01849

53. Perez-Rodriguez M, Biester H, Aboal JR *et al.* Thawing of snow and ice caused extraordinary high and fast mercury fluxes to lake sediments in Antarctica. *Geochim Cosmochim Acta*. 2019; **248**: 109-122. doi: 10.1016/j.gca.2019.01.009

54. Sun X, Zhang Q, Zhang G *et al.* Melting Himalayas and mercury export: Results of continuous observations from the Rongbuk Glacier on Mt. Everest and future insights. *Water Res*. 2022; **218**: 118474. doi: 10.1016/j.watres.2022.118474

55. Verbeke BA, Lamit LJ, Lilleskov EA *et al.* Latitude, elevation, and mean annual temperature predict peat organic matter chemistry at a global scale. *Global Biogeochemical Cycles*. 2022; **36**(2): e2021GB007057. doi: 10.1029/2021GB007057

56. Biester H, Martinez-Cortizas A, Birkenstock S *et al.* Effect of peat decomposition and mass loss on historic mercury records in peat bogs from Patagonia. *Environ Sci Technol*. 2003; **37**(1): 32-39.

57. Sirota JI, Kolka RK, Sebestyen SD *et al.* Mercury dynamics in the pore water of peat columns during experimental freezing and thawing. *Journal of Environmental Quality*. 2020; **49**(2): 404-416. doi: 10.1002/jeq2.20046

58. Martı́nez-Cortizas A, Pontevedra-Pombal X, Garcı́a-Rodeja E *et al.* Mercury in a Spanish Peat Bog: Archive of Climate Change and Atmospheric Metal Deposition. *Science*. 1999; **284**(5416): 939-942. doi: doi:10.1126/science.284.5416.939

59. Yee YH, Bishop K, Pettersson C *et al.* SUBCATCHMENT OUTPUT OF MERCURY AND METHYLMERCURY AT SVARTBERGET IN NORTHERN SWEDEN. *Water, Air, and Soil Pollution*. 1995; **80**(1-4): 455-465.

60. Thiagarajan N, McManus JF. Productivity and sediment focusing in the Eastern Equatorial Pacific during the last 30,000 years. *Deep Sea Research Part I: Oceanographic Research Papers*. 2019; **147**: 100-110. doi: 10.1016/j.dsr.2019.03.007

61. Martin J, Sanchez-Cabeza JA, Eriksson M *et al.* Recent accumulation of trace metals in sediments at the DYFAMED site (Northwestern Mediterranean Sea). *Mar Pollut Bull*. 2009; **59**(4-7): 146-153. doi: 10.1016/j.marpolbul.2009.03.013

62. Cossa D, Knoery J, Bănaru D *et al.* Mediterranean Mercury Assessment 2022: An Updated Budget, Health Consequences, and Research Perspectives. *Environ Sci Technol*. 2022; **56**(7): 3840-3862. doi: 10.1021/acs.est.1c03044

63. Lamborg CH, Hammerschmidt CR, Bowman KL *et al.* A global ocean inventory of anthropogenic mercury based on water column measurements. *Nature*. 2014; **512**(7512): 65-68. doi: 10.1038/nature13563

64. Weigelt A, Ebinghaus R, Pirrone N *et al.* Tropospheric mercury vertical profiles between 500 and 10 000 m in central Europe. *Atmospheric Chemistry and Physics*. 2016; **16**(6): 4135-4146. doi: 10.5194/acp-16-4135-2016

65. Yang H, Battarbee RW, Turner SD *et al.* Historical Reconstruction of Mercury Pollution Across the Tibetan Plateau Using Lake Sediments. *Environ Sci Technol*. 2010; **44**(8): 2918-2924. doi: 10.1021/es9030408

66. Zheng J. Archives of total mercury reconstructed with ice and snow from Greenland and the Canadian High Arctic. *Sci Total Environ*. 2015; **509**: 133-144.

67. Guo D, Yu E, Wang H. Will the Tibetan Plateau warming depend on elevation in the future? *Journal of Geophysical Research: Atmospheres*. 2016; **121**(8): 3969-3978.

68. Pepin N, Deng H, Zhang H *et al.* An Examination of Temperature Trends at High Elevations Across the Tibetan Plateau: The Use of MODIS LST to Understand Patterns of Elevation-Dependent Warming. *Journal of Geophysical Research: Atmospheres*. 2019; **124**(11): 5738-5756. doi: 10.1029/2018JD029798

69. Khan A, Haque SM, Biswas B. Altitudinal Shifting of Apple Orchards with Adaption of Changing Climate in the Alpine Himalaya. *Journal of the Indian Society of Remote Sensing*. 2023; **51**(5): 1135-1155. doi: 10.1007/s12524-023-01678-0

70. Sanz‐Elorza M, Dana ED, González A *et al.* Changes in the High‐mountain Vegetation of the Central Iberian Peninsula as a Probable Sign of Global Warming. *Ann Bot*. 2003; **92**(2): 273-280. doi: 10.1093/aob/mcg130

71. Streets DG, Horowitz HM, Lu Z *et al.* Global and regional trends in mercury emissions and concentrations, 2010–2015. *Atmos Environ*. 2019; **201**: 417-427. doi: 10.1016/j.atmosenv.2018.12.031

72. Verbrugge B, Geenen S. Global Gold Production Touching Ground: Expansion, Informalization, and Technological Innovation. Cham: Cham: Springer International Publishing; 2020.

73. Biester H, Bindler R, Martinez-Cortizas A *et al.* Modeling the Past Atmospheric Deposition of Mercury Using Natural Archives. *Environ Sci Technol*. 2007; **41**(14): 4851-4860. doi: 10.1021/es0704232

74. Chellman N, McConnell JR, Arienzo M *et al.* Reassessment of the Upper Fremont Glacier Ice-Core Chronologies by Synchronizing of Ice-Core-Water Isotopes to a Nearby Tree-Ring Chronology. *Environ Sci Technol*. 2017; **51**(8): 4230-4238. doi: 10.1021/acs.est.6b06574

75. Schuster PF, Krabbenhoft DP, Naftz DL *et al.* Atmospheric mercury deposition during the last 270 years: A glacial ice core record of natural and anthropogenic sources. *Environ Sci Technol*. 2002; **36**(11): 2303-2310. doi: 10.1021/es0157503

76. Janssens-Maenhout G, Pagliari V, Guizzardi D *et al.* Global emission inventories in the Emission Database for Global Atmospheric Research (EDGAR)–Manual (I). *Gridding: EDGAR emissions distribution on global gridmaps, Publications Office of the European Union, Luxembourg*. 2013; **775**.

77. Rossmann R. Protocol to Reconstruct Historical Contaminant Loading to Large Lakes: The Lake Michigan Sediment Record of Mercury. *Environ Sci Technol*. 2010; **44**(3): 935-940. doi: 10.1021/es902307c

78. Fitzgerald WF, Engstrom DR, Lamborg CH *et al.* Modern and historic atmospheric mercury fluxes in northern Alaska: Global sources and Arctic depletion. *Environ Sci Technol*. 2005; **39**(2): 557-568.

79. Martin A. *Uncovered The dark world of the Zama Zamas*. 2019.

80. Grynberg R, Singogo F. The ASGM Sector in Africa: Lessons from China. 2021. 49-109.

81. AMAP/UNEP. *Technical Background Report for the Global Mercury Assessment 2018*. Arctic Monitoring and Assessment Programme, Oslo, Norway/UN Environment Programme, Chemicals and Health Branch, Geneva, Switzerland; 2019.

82. Rose NL, Milner AM, Fitchett JM *et al.* Natural archives of long-range transported contamination at the remote lake Letšeng-la Letsie, Maloti Mountains, Lesotho. *Sci Total Environ*. 2020; **737**: 139642.

83. Kirk JL, St. Louis VL, Sharp MJ. Rapid reduction and reemission of mercury deposited into snowpacks during atmospheric mercury depletion events at Churchill, Manitoba, Canada. *Environ Sci Technol*. 2006; **40**(24): 7590-7596.

84. Moore CW, Obrist D, Steffen A *et al.* Convective forcing of mercury and ozone in the Arctic boundary layer induced by leads in sea ice. *Nature*. 2014; **506**(7486): 81-84. doi: 10.1038/nature12924

85. Steffen A, Bottenheim J, Cole A *et al.* Atmospheric mercury over sea ice during the OASIS-2009 campaign. *Atmospheric Chemistry and Physics*. 2013; **13**(14): 7007-7021. doi: 10.5194/acp-13-7007-2013

86. Kang S, Huang J, Wang F *et al.* Atmospheric Mercury Depositional Chronology Reconstructed from Lake Sediments and Ice Core in the Himalayas and Tibetan Plateau. *Environ Sci Technol*. 2016; **50**(6): 2859-2869. doi: 10.1021/acs.est.5b04172

87. Norton SA, Jacobson GL, Kopacek J *et al.* A comparative study of long-term Hg and Pb sediment archives. *Environmental Chemistry*. 2016; **13**(3): 517-527. doi: 10.1071/En15114

88. Rausch N, Nieminen TM, Ukonmaanaho L *et al.* Retention of atmospheric Cu, Ni, Cd and Zn in an ombrotrophic peat profile near the Outokumpu Cu-Ni mine, SE-Finland. *JOURNAL DE PHYSIQUE IV*. 2003; **107**: 1127-1130. doi: 10.1051/jp4:20030499

89. Moore TR, Bubier JL, Heyes A *et al.* Methyl and Total Mercury in Boreal Wetland Plants, Experimental Lakes Area, Northwestern Ontario. *Journal of Environmental Quality*. 1995; **24**(5): 845-850. doi: <https://doi.org/10.2134/jeq1995.00472425002400050007x>

90. Miszczak E, Stefaniak S, Michczynski A *et al.* A novel approach to peatlands as archives of total cumulative spatial pollution loads from atmospheric deposition of airborne elements complementary to EMEP data: priority pollutants (Pb, Cd, Hg). *Sci Total Environ*. 2020; **705**: 135776. doi: 10.1016/j.scitotenv.2019.135776

91. Norton SA, Evans GC, Kahl JS. Comparison of Hg and Pb Fluxes to Hummocks and Hollows of Ombrotrophic Big Heath Bog and to Nearby Sargent Mt. Pond, Maine, USA. *Water, Air, and Soil Pollution*. 1997; **100**(3): 271-286. doi: 10.1023/A:1018380610893

92. Ettler V, Navrátil T, Mihaljevič M *et al.* Mercury deposition/accumulation rates in the vicinity of a lead smelter as recorded by a peat deposit. *Atmos Environ*. 2008; **42**(24): 5968-5977. doi: <https://doi.org/10.1016/j.atmosenv.2008.03.047>

93. Wasik JKC, Engstrom DR, Mitchell CPJ *et al.* The effects of hydrologic fluctuation and sulfate regeneration on mercury cycling in an experimental peatland. *Journal of Geophysical Research: Biogeosciences*. 2015; **120**(9): 1697-1715. doi: 10.1002/2015JG002993

94. Osterwalder S, Bishop K, Alewell C *et al.* Mercury evasion from a boreal peatland shortens the timeline for recovery from legacy pollution. *Scientific Reports*. 2017; **7**(1): 16022. doi: 10.1038/s41598-017-16141-7

95. Fritsche J, Osterwader S, Nilsson MB *et al.* Evasion of Elemental Mercury from a Boreal Peat land Suppressed by Long-Term Sulfate Addition. *Environmental Science & Technology Letters*. 2014; **1**(10): 421-425. doi: 10.1021/ez500223a

96. Novak M, Stepanova M, Jackova I *et al.* Isotopic evidence for nitrogen mobility in peat bogs. *Geochim Cosmochim Acta*. 2014; **133**: 351-361. doi: 10.1016/j.gca.2014.02.021

97. Bandara S, Froese DG, St Louis VL *et al.* Postdepositional Mercury Mobility in a Permafrost Peatland from Central Yukon, Canada. *ACS Earth and Space Chemistry*. 2019; **3**(5): 770-778. doi: 10.1021/acsearthspacechem.9b00010

98. Korosi JB, Griffiths K, Smol JP *et al.* Trends in historical mercury deposition inferred from lake sediment cores across a climate gradient in the Canadian High Arctic. *Environ Pollut*. 2018; **241**: 459-467. doi: <https://doi.org/10.1016/j.envpol.2018.05.049>

99. Evenset A, Christensen G, Carroll J *et al.* Historical trends in persistent organic pollutants and metals recorded in sediment from Lake Ellasjøen, Bjørnøya, Norwegian Arctic. *Environ Pollut*. 2007; **146**(1): 196-205. doi: 10.1016/j.envpol.2006.04.038

100. Kamman NC, Engstrom DR. Historical and present fluxes of mercury to Vermont and New Hampshire lakes inferred from 210Pb dated sediment cores. *Atmos Environ*. 2002; **36**(10): 1599-1609. doi: <https://doi.org/10.1016/S1352-2310(02)00091-2>

101. Ribeiro Guevara S, Meili M, Rizzo A *et al.* Sediment records of highly variable mercury inputs to mountain lakes in Patagonia during the past millennium. *Atmospheric Chemistry and Physics*. 2010; **10**(7): 3443-3453. doi: 10.5194/acp-10-3443-2010

102. Shotbolt LA, Thomas AD, Hutchinson SM. The use of reservoir sediments as environmental archives of catchment inputs and atmospheric pollution. *Progress in Physical Geography*. 2005; **29**(3): 337-361.

103. Roberts SL, Kirk JL, Muir DCG *et al.* Quantification of Spatial and Temporal Trends in Atmospheric Mercury Deposition across Canada over the Past 30 Years. *Environ Sci Technol*. 2021; **55**(23): 15766-15775. doi: 10.1021/acs.est.1c04034

104. Wiklund JA, Kirk JL, Muir DCG *et al.* Anthropogenic mercury deposition in Flin Flon Manitoba and the Experimental Lakes Area Ontario (Canada): A multi-lake sediment core reconstruction. *Sci Total Environ*. 2017; **586**: 685-695. doi: 10.1016/j.scitotenv.2017.02.046

105. Lent RM, Alexander CR. Mercury accumulation in Devils Lake, North Dakota — Effects of environmental variation in closed-basin lakes on mercury chrologies. *Water, Air, and Soil Pollution*. 1997; **98**(3): 275-296. doi: 10.1007/BF02047039

106. Fitzgerald WF, Engstrom DR, Lamborg CH *et al.* Modern and historic atmospheric mercury fluxes in northern Alaska: Global sources and Arctic depletion. *Environ Sci Technol*. 2005; **39**(2): 557-568. doi: 10.1021/es049128x

107. Fitzgerald WF, Engstrom DR, Lamborg CH *et al.* Modern and Historic Atmospheric Mercury Fluxes in Northern Alaska:  Global Sources and Arctic Depletion. *Environ Sci Technol*. 2005; **39**(2): 557-568. doi: 10.1021/es049128x

108. Lehman JT. Reconstructing the Rate of Accumulation of Lake Sediment: The Effect of Sediment Focusing1. *Quatern Res*. 1975; **5**(4): 541-550.

109. Liu MD, Zhang QR, Maavara T *et al.* Rivers as the largest source of mercury to coastal oceans worldwide. *Nature Geoscience*. 2021; **14**(9): 672-677. doi: 10.1038/s41561-021-00793-2

110. Dastoor A, Angot H, Bieser J *et al.* Arctic mercury cycling. *Nature Reviews Earth & Environment*. 2022; **3**(4): 270-286. doi: 10.1038/s43017-022-00269-w

111. Asmund G, Nielsen SP. Mercury in dated Greenland marine sediments. *Sci Total Environ*. 2000; **245**(1): 61-72. doi: 10.1016/S0048-9697(99)00433-7

112. Abril G, Commarieu MV, Sottolichio A *et al.* Turbidity limits gas exchange in a large macrotidal estuary. *Estuarine Coastal and Shelf Science*. 2009; **83**(3): 342-348. doi: 10.1016/j.ecss.2009.03.006

113. Outridge PM, Mason R, Wang F *et al.* Updated global and oceanic mercury budgets for the United Nations Global Mercury Assessment 2018. *Environ Sci Technol*. 2018; **52**(20): 11466-11477.

114. Zhang Y, Jaeglé L, Thompson L *et al.* Six centuries of changing oceanic mercury. *Global Biogeochemical Cycles*. 2014; **28**(11): 1251-1261. doi: 10.1002/2014GB004939

115. USGS. Space Shuttle Radar Topography Mission (SRTM). 2000.

116. Bosilovich MG. *MERRA-2: Initial evaluation of the climate*: National Aeronautics and Space Administration, Goddard Space Flight Center, 2015.

117. Koster RD, McCarty W, Coy L *et al.* *MERRA-2 input observations: Summary and assessment*. 2016.

118. Muntean M, Janssens-Maenhout G, Song S *et al.* Trend analysis from 1970 to 2008 and model evaluation of EDGARv4 global gridded anthropogenic mercury emissions. *Sci Total Environ*. 2014; **494-495**: 337-350. doi: 10.1016/j.scitotenv.2014.06.014

119. bp. bp Statistical Review of World Energy - all data. 2022.

120. World Bank. GNI per capita, Atlas method (current US$). 2022.
